# Active site specificity profiling datasets of matrix metalloproteinases (MMPs) 1, 2, 3, 7, 8, 9, 12, 13 and 14

**DOI:** 10.1016/j.dib.2016.02.036

**Published:** 2016-02-22

**Authors:** Ulrich Eckhard, Pitter F. Huesgen, Oliver Schilling, Caroline L. Bellac, Georgina S. Butler, Jennifer H. Cox, Antoine Dufour, Verena Goebeler, Reinhild Kappelhoff, Ulrich auf dem Keller, Theo Klein, Philipp F. Lange, Giada Marino, Charlotte J. Morrison, Anna Prudova, David Rodriguez, Amanda E. Starr, Yili Wang, Christopher M. Overall

**Affiliations:** a Centre for Blood Research, Department of Oral Biological and Medical Sciences, University of British Columbia, Vancouver, BC, Canada; b Department of Biochemistry and Molecular Biology, University of British Columbia, Vancouver, BC, Canada

**Keywords:** Matrix metalloproteinases, MMPs, PICS, Proteomics, Quenched fluorescence, Specificity profiling, Cleavage sites

## Abstract

The data described provide a comprehensive resource for the family-wide active site specificity portrayal of the human matrix metalloproteinase family. We used the high-throughput proteomic technique PICS (Proteomic Identification of protease Cleavage Sites) to comprehensively assay 9 different MMPs. We identified more than 4300 peptide cleavage sites, spanning both the prime and non-prime sides of the scissile peptide bond allowing detailed subsite cooperativity analysis. The proteomic cleavage data were expanded by kinetic analysis using a set of 6 quenched-fluorescent peptide substrates designed using these results. These datasets represent one of the largest specificity profiling efforts with subsequent structural follow up for any protease family and put the spotlight on the specificity similarities and differences of the MMP family. A detailed analysis of this data may be found in Eckhard et al. (2015) [1]. The raw mass spectrometry data and the corresponding metadata have been deposited in PRIDE/ProteomeXchange with the accession number **PXD002265**.

Specifications Table

**Table:** 

Subject area	Biology
More specific subject area	Proteolytic enzymes, metalloproteinases, substrate specificity profiling, inhibitor design, drug discovery, matrix biology, extra cellular matrix (ECM).
Type of data	Mass spectrometry raw-files; search engine output files; metadata; quenched fluorescent peptide cleavage data; specificity profiling analysis (.xlsx).
How data was acquired	Liquid chromatography tandem mass spectrometry (LC-MS/MS): either QSTAR XL or QSTAR Pulsar I (Applied Biosystems) mass spectrometer coupled on-line to LC Packings capillary LC system (Dionex).
Data format	RAW files: .wiff and .mzXML-files; .tandem and .pepxml post-database search output files from X! Tandem [2].
Experimental factors	(A) Human MMPs 1, 2, 3, 8, 9 and 13 were expressed and purified from CHO or *Timp2*^*−/−*^ MEF-conditioned medium [3], [4], [5], [6], [7]. Soluble MMP14 (A21-R513) was purified from *Pichia pastoris*[3]. MMP7 was purchased from Enzo Life Sciences and MMP12 was a kind gift from Novartis. ProMMP3 was activated using chymotrypsin; all other proMMPs were activated using APMA. (B) Proteome-derived peptide libraries were prepared from K562 cells in the presence of protease inhibitors [8], [9]. Proteins were denatured and cysteine side-chains alkylated. After reaction clean-up, proteomes were digested with trypsin or GluC giving orthogonal peptide libraries. Primary amines were dimethylated, peptide libraries purified, and stored as 200 µg aliquots at −80 °C.
Experimental features	PICS cleavage assays [8], [9] were performed by incubating peptide libraries with MMP. Cleaved peptides with neo-N-termini were biotinylated and affinity purified. Eluates were desalted and analyzed by LC–MS/MS. Spectra were matched to peptides using X! Tandem [2] and statistically evaluated with PeptideProphet [10], [11]. Identified peptides represent prime-side cleavage products and complete cleavage sites were reconstructed using WebPICS [12].
Data source location	Overall Laboratory, Centre for Blood Research, Department of Oral Biological and Medical Sciences, Faculty of Dentistry, University of British Columbia, Vancouver, BC, Canada. 49 °15′44.5″N 123 °14′41.8″W.
Data accessibility	The mass spectrometry raw data have been deposited in PRIDE/ProteomeXchange with the accession number **PXD002265**.

Value of the data

• Comprehensive specificity profiling data of nine matrix metalloproteinases using PICS proteomics.
• Largest compendium of matrix metalloproteinase P6–P6′ cleavage sites, reporting more than 4300 cleaved peptides.
• Identified cleavage sites allow in-depth cooperativity analysis of specificity subsites.
• Quenched fluorescent peptide cleavage data corroborating the specificity profiles.
• These data can guide small molecule drug development and help in discrimination between direct and indirect MMP targets.

## 1 Data

Matrix metalloproteinases (MMPs) regulate the structural matrix environment and extracellular signaling by precise proteolytic cleavage. Unraveling complex *in vivo* proteolytic networks is challenging. Thus comprehensive specificity profiles of all proteases involved are needed to guide interpretation.

The data in the ProteomeXchange archive (**PXD002265**) and accompanying data of the present article provide a comprehensive resource for the individual assessment of the active site specificity of nine representative members of the human matrix metalloproteinase (MMP) family. The in-depth specificity comparison based on these proteomic data corroborated with kinetic analysis using a set of 6 quenched fluorescent peptides and *in silico* peptide docking was presented recently [1].

MMPs 1, 2, 3, 7, 8, 9, 12, 13 and 14 were all assayed by the high-throughput Proteomic Identification of protease Cleavage Sites (PICS; or PICS proteomics) method [8], [9] and using two orthogonal human whole-proteome peptide libraries (generated with trypsin or GluC). These data were analyzed using X! Tandem [2] for peptide-spectrum matching, and PeptideProphet [11] for statistical evaluation. However, other search engines such as Mascot [13], MS-GF+ [14], Comet [15], or MS Amanda [16] can be used to extend the number of matched spectra by combining the results using *e.g.* iProphet [17] within the Trans Proteomic Pipeline [10] or PeptideShaker [18]. Here we provide additional data to enable other researchers to (i) reinvestigate our analysis to identify additional subsite cooperativity effects and so far unexplored specificity preferences, and (ii) reanalyze our raw-data with entirely new concepts or ideas in mind, such as specificity-oriented protease evolution [19], [20], functional phylogeny [21], or substrate-driven mapping of the degradome [22].

**Fig. 1:**
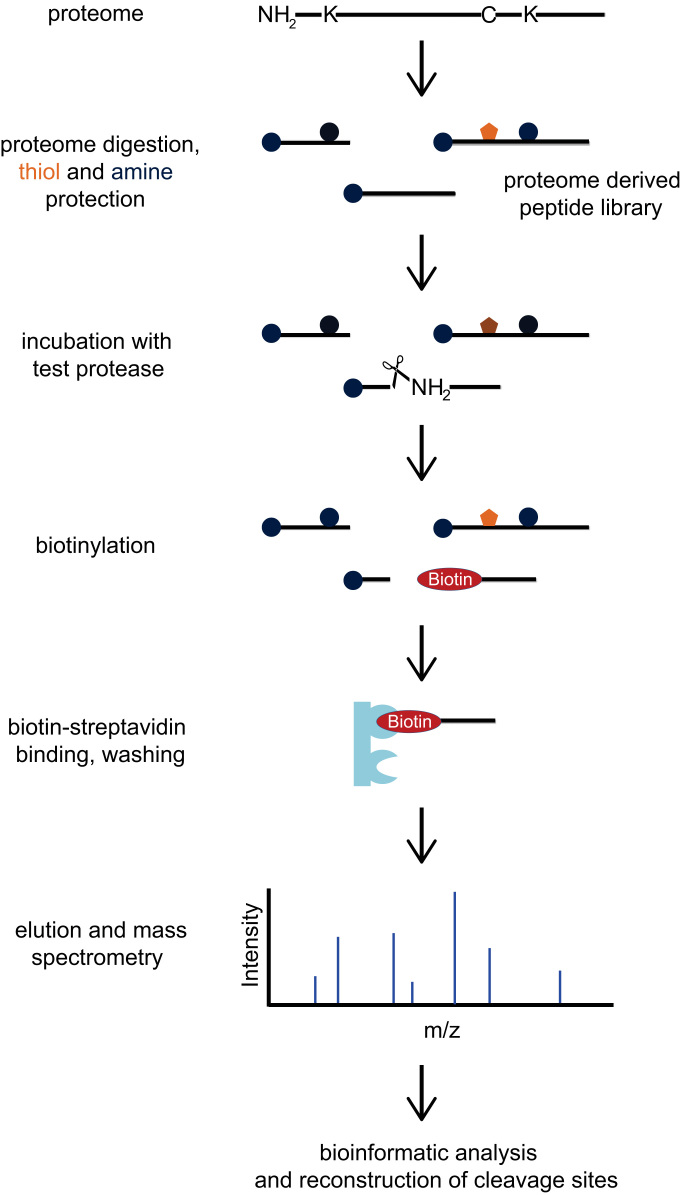
Proteomic Identification of protease Cleavage Sites (PICS) workflow. PICS libraries are generated from cellular proteomes, *e.g.* human cell cultures, and thus represent biological sequence diversity. Specific endoproteases such as trypsin, GluC, or chymotrypsin are then used to digest proteomes into peptides amenable for mass spectrometry. Primary amines and thiols are chemically protected and the peptide library is purified. Next, the proteome-derived peptide library is incubated with the endoprotease of interest (*e.g.* MMP1). Prime side cleavage products (*i.e.* peptide fragments C-terminal of the cleavage site depicted between P1 and P1′) possess free and thus reactive N-termini that are subsequently tagged with cleavable biotin allowing specific isolation with immobilized streptavidin. Following elution, prime-side cleavage products are identified by LC–MS/MS, and the corresponding non-prime sequences are reconstructed bioinformatically, *e.g.* by using the free webservice WebPICS (http://clipserve.clip.ubc.ca/pics/) [12].

**Fig. 2:**
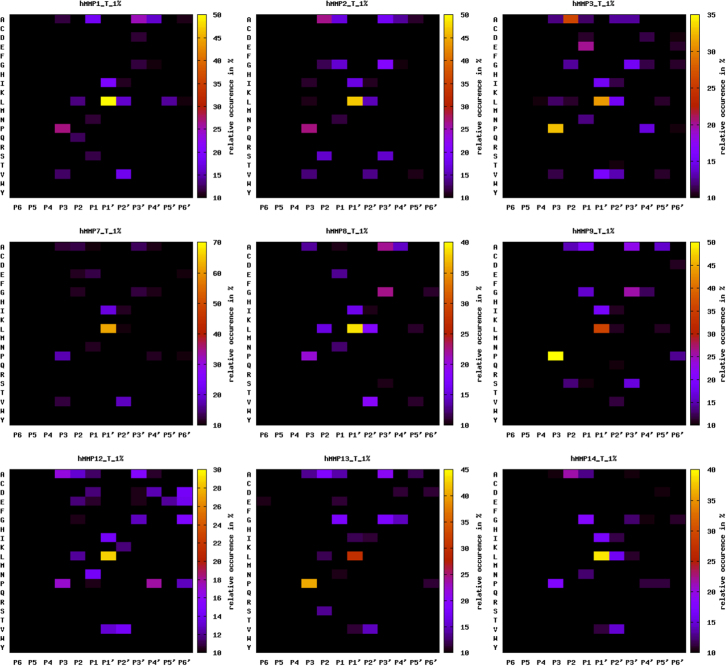
Sequence specificity profiles of MMPs 1, 2, 3, 7, 8, 9, 12, 13, and 14 using trypsin-generated human peptide libraries. Identified cleavage sites are summarized as heat maps showing relative amino acid occurrence. P6–P6′ subsite positions are shown on the *x* axes with the identified cleavage site between P1 and P1′. Plotted amino acids are indicated on the *y* axes with single-letter codes. Please refer to Supplementary Fig S2 for corresponding results using GluC-generated peptide libraries.

**Fig. 3:**
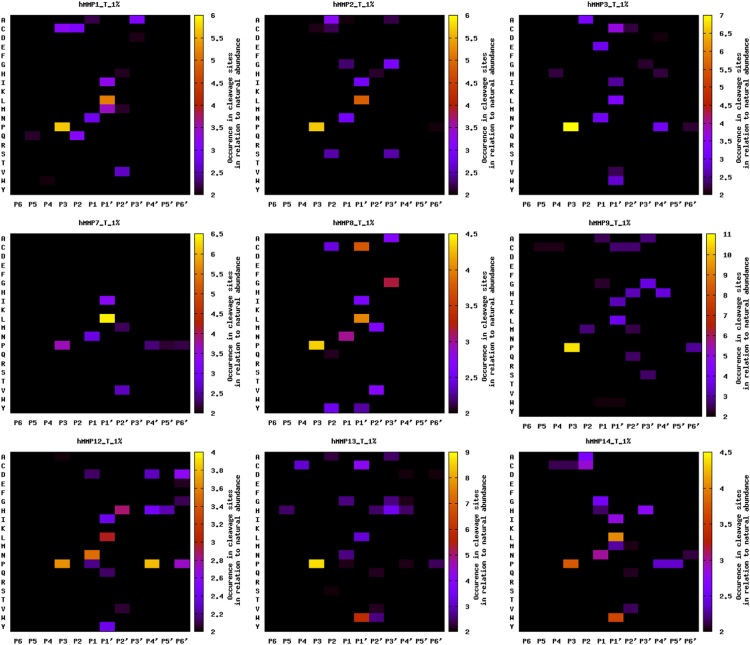
MMP sequence specificity profiles identified in trypsin-generated human peptide libraries and summarized as heat maps showing fold-change over natural abundance. P6–P6′ subsite positions are shown on the x axes with the identified cleavage site between P1 and P1′. Plotted amino acids are indicated on the y axes with single-letter codes. Please refer to Supplementary Fig S3 for corresponding results using GluC-generated peptide libraries.

A representative PICS workflow is depicted in Fig. 1, and a graphical representation of the various MMP domain architectures is shown in Supplementary Fig S1. Specificity profiling results were summarized as heat maps in Fig. 2, Fig. 3 (trypsin generated libraries) and 2.1.8, 2.1.8 (GluC-generated libraries). Supplementary Table S1 provides a general resource for the MMPs analyzed, giving their domain boundaries and links to the major databases in protein and protease research: UniProt [23], Pfam [24], MEROPS [25], and TopFIND [26], [27], [28]. Supplementary Table S2 depicts key residues and all secondary structure elements characterizing the different MMP catalytic domains, and shows a structural overlay of all analyzed MMPs. Supplementary Tables 3 and 4 contain the individual MMP specificity profiling results obtained from trypsin- and Glu-generated peptide libraries, respectively. Identified thioacylated prime-side cleavage products (column A), the WebPICS [12] results (columns C–I) including HeatMaps (www.gnuplot.info) and iceLogos [29], and the individual subsite analysis (columns K–W) together with the amino acid distribution in the human proteome (UniProt release 2013_10) used for calculating normalized abundances (columns Y–AA) are shown. Additionally, a high resolution PICS workflow is shown on both index pages, and all original WebPICS results are provided in the supplement as a combined zip-file. Supplementary Table S5 provides the data matrices underlying our subsite cooperativity analysis, in which we fixed certain subsites (*e.g.* P3-Pro or P1′-Leu) and analyzed the changes of the individual amino acid occurrences in the other subsites (P6–P6′). Supplementary Table S6 provides the raw data of our quenched fluorescent cleavage assays, including a graphical representation of two of the peptides (PLG↓L and PAN↓L) and a high resolution table summarizing the results normalized to the standard MMP substrate PLG↓L (originally referred to as QF-24) [30]. Detailed database search settings for PICS data analysis are given in Supplementary Table S7. Additionally, a combined zip-file of all WebPICS results can be found in the supplement. All mass spectrometry raw data and corresponding metadata have been deposited in the ProteomeXchange Consortium database (http://proteomecentral.proteomexchange.org) via the PRIDE partner repository [51] with the PXD identifier 〈**PXD002265**〉.

We previously used PICS to characterize a wide selection of different proteases, such as clostridial collagenases [31], plant metalloproteinases [32], human type II transmembrane serine proteases (TTSPs) [33], the *archaeal* protease LysargiNase [34], snake venom serine proteinases [35], human coagulation factor Xa [12], and caspases 3 and 7 [8], proving both its versatility and robustness. Importantly, PICS is designed for active site specificity profiling and not for the identification of native substrates. For the latter task, the Overall lab developed TAILS (Terminal Amine Isotopic Labeling of Substrates) [36], [37], which we have successfully used *e.g.* for the identification of natural substrates of dipeptidyl peptidases 8 and 9 [38], the human gelatinases MMP2 and 9 [39], membrane-type 6 matrix metalloprotease MMP25 [40] and the meprins [41]. Over the last few years we have adapted TAILS for the study of complex *in vivo* biological systems [42] and identification of N-terminal modifications such as proteolytic processing that alter protein stability or function [43], [44]. We have assessed the N-terminome of various tissues and cells, such as skin [42], erythrocytes [43], platelets [45], and dental pulp [46]. In combination, TAILS N-terminomics and PICS proteomics allow an in depth characterization of any biological system and protease in an unbiased, proteomics-centered manner.

The following materials and methods section will enable other investigators and laboratories to design similar experimental procedures to study matrix metalloproteinases or any other protease by PICS proteomics. Please refer to our recent dental pulp proteomics and N-terminomics Data in Brief article for more information on TAILS [47].

## 2 Experimental design, materials and methods

### 2.1 Expression and purification of human MMPs

#### 2.1.1 **Summary**

MMPs 1, 2, 3, 8, 9 and 13 were expressed as zymogens using the pGW1HG vector (kindly provided by British Biotech Pharmaceuticals, Oxford, UK), and purified from serum-free conditioned medium from (i) Chinese hamster ovary (CHO) cells or (ii) murine embryonic *Timp2*^*−/*^^−^ fibroblasts (MEFs) [3], [4], [5], [6], [7]. Soluble MMP14 lacking the C-terminal transmembrane and cytoplasmic domain (A21-R513) was purified from *Pichia pastoris* [3]. Purified proMMPs were aliquoted into single use aliquots of 10 μg, flash-frozen with liquid nitrogen, and stored at −70 °C until use. For more expression and purification details, see below. Active MMP7 was purchased from Enzo Life Sciences, and MMP12 was a kind gift from Novartis Pharma AG (Basel, CH). ProMMP3 was activated using chymotrypsin. All others proMMPs were activated using APMA.

#### 2.1.2 **ProMMP1**

Chinese hamster ovary (CHO-K1) cells (American Type Culture Collection) were maintained in Dulbecco׳s modified Eagle׳s medium (Invitrogen) supplemented with 10% cosmic calf serum (HyClone Laboratories, Inc.) and non-essential amino acids (Invitrogen). Cells were transfected with pGW1HG-MMP1 and selected with 25 μg/ml mycophenolic acid (Invitrogen). MMP1-expressing clones were expanded to confluence in roller bottles (850 cm^2^, BD Biosciences), washed with phosphate-buffered saline (PBS; 138 mm NaCl, 2.7 mm KCl, 20 mm Na_2_HPO4, 1.5 mm KH_2_PO4, pH 7.4), and incubated in 100 ml of serum-free CHO-S-SFM II medium (Invitrogen). Conditioned serum-free medium was collected every 1–2 days for up to 8 days. ProMMP1 was purified from collected culture supernatants using an (i) Orange-Sepharose column equilibrated in MES buffer (50 mM MES, pH 6.0, 5 mm CaCl_2_, 0.1 M NaCl, 0.025% sodium azide), and eluted with 1 M NaCl (in MES buffer). Elution fractions were subsequently loaded on (ii) Zn^2+^-chelating Sepharose Fast Flow resin (Amersham Biosciences), and chromatographed with a linear imidazole gradient (0 to 0.5 M). Fractions containing proMMP1 were pooled and dialyzed into HEPES buffer (50 mM HEPES pH 7.2, 5 mM CaCl_2_, 0.1 M NaCl).

#### 2.1.3 **ProMMP2**

TIMP-2-free human proMMP2 was expressed in ras/myc-transformed *Timp2*^−/−^ fibroblasts. Cells were grown in Dulbecco׳s modified Eagle׳s medium with 10% cosmic calf serum (HyClone Laboratories Inc), transfected with MMP2-pGW1HG, and selected by using 25 μg/ml mycophenolic acid. Serum-free conditioned medium was harvested from roller bottles and proMMP2 was purified at 4 °C in MES buffer by (i) gelatin-Sepharose (Amersham Biosciences) chromatography. After elution with 10% dimethyl sulfoxide in HEPES buffer, samples were dialyzed into MES buffer, and loaded in tandem onto (ii) lentil lectin-Sepharose (Sigma) to remove MMP9 and fibronectin, and (iii) a 1 ml gelatin-Sepharose column to capture proMMP2. After elution using 10% dimethyl sulfoxide (gelatin-Sepharose column only), fractions containing proMMP2 were pooled and dialyzed into MES buffer.

#### 2.1.4 **ProMMP3**

Recombinant C-terminally FLAG-tagged human proMMP3 was expressed from pGW1HG in CHO-K1 cells and purified from supernatants in MES buffer using a (i) Green-agarose (Sigma) column. After elution with 1 M NaCl, eluates were loaded on a (ii) Zn^2+^-chelating column (Amersham Biosciences) and chromatographed with an imidazole gradient. Fractions containing proMMP3 were pooled, dialyzed into Tris-buffered saline (TBS; 50 mM Tris, 150 mM NaCl, pH 7.4), and subsequently loaded onto an (iii) anti-FLAG-agarose column (Sigma). After elution with 100 mM glycine, pH 3.5, fractions were immediately adjusted to pH 7–8 using 1 M Tris pH 8.0, and fractions containing proMMP3 were pooled and dialyzed into HEPES buffer.

#### 2.1.5 **ProMMP8**

CHO-K1 cells were transfected with pGW1HG-MMP8 and selected using 25 μg/ml mycophenolic acid (Invitrogen); conditioned medium was collected from roller bottles. To remove gelatinases (MMP2 and MMP9), culture supernatants were chromatographed over (i) gelatin-Sepharose 4B resin (Amersham Biosciences) connected in tandem with (ii) a Red-Sepharose CL-6B column (Amersham Biosciences). MMP8 was eluted from Red-Sepharose with 1 M NaCl in TBS, and (iii) loaded on a column of Zn^2+^-chelating Sepharose resin (Amersham Biosciences). Fractions containing MMP-8 were pooled and chromatographed over (iv) lentil lectin-agarose-Sepharose 4B (Sigma-Aldrich) and eluted with 100 mm α-D-methylmannopyranoside (Sigma) in TBS. Purified proMMP8 was buffer exchanged into collagenase assay buffer (50 mM Tris, 200 mM NaCl, 5 mM CaCl_2_, 0.05% Brij-35, pH 7.4) using a PD-10 Sephadex G-25 column (Amersham Biosciences).

#### 2.1.6 **ProMMP9**

Human MMP9 was expressed from pGW1HG in CHO-K1 cells with 25 μg/ml mycophenolic acid for selection. ProMMP9 was captured from conditioned medium on a gelatin-Sepharose column (Amersham Biosciences) in MES buffer, and eluted after extensive washing in MES buffer supplemented with 10% dimethyl sulfoxide. ProMMP-9 was dialyzed into HEPES buffer.

#### 2.1.7 **ProMMP13**

Recombinant C-terminally FLAG-tagged human MMP13 was expressed in CHO-K1 cells from pGW1HG and purified from culture supernatants using a (i) green-agarose column (Sigma). After extensive washing with MES buffer, bound protein was eluted using 1 M NaCl, and fractions containing MMP-13 were dialyzed into TBS. MMP13 was then purified to homogeneity using an (ii) anti-FLAG-agarose column (Sigma). After elution with 100 mM glycine (pH 3.5), fractions were immediately adjusted to pH 7–8 using 1 M Tris pH 8.0. ProMMP13 was dialyzed into HEPES buffer.

#### 2.1.8 **ProMMP14**

Soluble MT1-MMP with a FLAG tag in place of the transmembrane and cytoplasmic domains was cloned into pPIC9 (Invitrogen) and expressed in Pichia GS115 cells (Invitrogen). Cells were grown in 500 ml baffled flasks. After 24 h, 0.5% methanol was added to induce recombinant protein expression. Culture medium was diluted in MES buffer and MT1-MMP was purified using a red agarose column (Sigma). After extensive washing with MES buffer, protein was eluted with 1 M NaCl, and fractions containing proMMP14 were pooled and dialyzed into HEPES buffer.

## 3 PICS peptide library preparation.

Human whole proteome-derived peptide libraries for MMP specificity profiling were prepared as described in great detail in Nature Protocols [9]: in brief, cell pellets were collected from human lymphoblast cell K562 cultures and lysed in 20 mM HEPES (pH 7.5) supplemented with 0.1% (w/v) SDS and protease inhibitors to prevent unwanted proteolysis (1×Roche cOmplete plus 1 mM PMSF and 10 mM EDTA). Cell debris was removed by centrifugation (26,000*g*, 1 h, 4 °C); soluble proteins were denatured using guanidine hydrochloride (4 M), and cysteine side-chains were reduced with 20 mM DTT (1 h, 37 °C). Free sulfhydryl groups were protected with 40 mM iodoacetamide (3 h, 20 °C) to avoid peptide crosslinking and reactions were stopped by adding more DTT (5 mM, 15 min, 20 °C). Reaction clean-up was performed using chloroform/methanol precipitation as described elsewhere [48], pellets were air-dried and re-suspended in 100 mM HEPES, 5 mM CaCl_2_, pH 7.5, and digested with TPCK-treated trypsin or GluC (*Staphylococcus aureus* protease V8, Worthington) at a protease to proteome ratio of 1:100 (w/w) overnight at 37 °C. Note, another protease often used for PICS library preparation is chymotrypsin. After inactivation of trypsin/GluC with 1 mM PMSF (30 min, 20 °C), undigested protein aggregates were removed by centrifugation (20,000*g*, 10 min, 4 °C). Primary amines of peptide N-termini (α-amines) and lysine side chains (ε-amines) were blocked by reductive dimethylation with 30 mM formaldehyde (CH_2_O) and 15 mM sodium cyanoborohydride (NaCNBH_3_, Sterogene) at 20 °C for 16 h overnight (pH 6–7). To ensure completeness of amine blocking, another 15 mM formaldehyde and 15 mM sodium cyanoborohydride were added and incubated for additional two hours. Samples were desalted by size exclusion chromatography using Sephadex G-10 columns (10 mM potassium phosphate buffer, pH 2.7, 10% (v/v) methanol), and after methanol removal by vacuum concentration (SpeedVac, Thermo), peptides were purified by reversed-phase chromatography on an ÄKTA™ high-performance liquid chromatography system (Äkta Explorer, GE Healthcare) using a RESOURCE RPC column (GE Healthcare); wash buffer contained 0.3% (v/v) formic acid, and samples were eluted in 80% (v/v) acetonitrile, both in HPLC-grade H_2_O. These PICS peptide libraries were concentrated by rotary evaporation under vacuum, re-suspended in water, and stored in 200–400 µg aliquots of 5–15 mg/ml at −80 °C until use. Peptide concentration was estimated using the bicinchoninic assay (BCA, Pierce). All reagents were purchased from Sigma-Aldrich unless otherwise specified.

## 4 PICS cleavage site specificity assay.

MMP cleavage assays were performed by incubation of 200–400 µg human whole-proteome peptide library with active recombinant MMP at a protease to peptide library ratio of 1:100 (w/w) in 50 mM HEPES, 150 mM NaCl, 5 mM CaCl_2_ at pH 7.4, overnight, and stopped by heat inactivation at 70 °C for 30 min. Prime-side cleavage products generated by MMP cleavage were subsequently isolated by positive enrichment using the biotin handle. In short, cleaved peptides with a free primary amine at the N-terminus generated by MMP activity were biotinylated by incubation with 0.5 mM Sulfo-NHS-SS-Biotin, an amine-reactive biotin with a redox-sensitive and thus cleavable disulfide linker (Thermo Scientific) for 2 h at 20 °C. Biotinylated prime-side cleavage products were separated from uncleaved peptides by affinity purification, incubating with 300 μl Streptavidin Sepharose slurry (GE Healthcare) for 2 h with mild agitation. After extensive washing (50 mM HEPES, pH 7.2), biotinylated peptides were eluted with 20 mM DTT (2 h, 20 °C), desalted using reversed-phase solid phase extraction (Sep-Pak C18, Waters) with binding and washing in 0.1% (v/v) formic acid and elution in 80% (v/v) acetonitrile, both in HPLC-grade H_2_O. Eluates were vacuum dried to near dryness using a SpeedVac concentrator (Thermo), brought to 10 μl with 0.1% formic acid, and stored at −80 °C until LC–MS/MS analysis.

## 5 LC–MS/MS, peptide spectrum matching, and data analysis

LC-MS/MS analysis was performed using an LC Packings capillary LC system (Dionex) coupled online to a quadrupole time-of-flight mass spectrometer operated either by the UBC Center for Blood Research Mass Spectrometry Suite (QSTAR XL; Applied Biosystems), or by the UBC Proteomics Core Facility (QSTAR Pulsar I, Applied Biosystems). Samples were diluted in 0.3% (v/v) formic acid and loaded onto a column packed with Magic C18 resin (Michrom Bioresources). Peptides were eluted using a 2–80% (v/v) acetonitrile gradient in 0.1% (v/v) formic acid over 95 min. MS/MS data were acquired automatically, using Analyst QS software, v1.1 (Applied Biosystems) for data-dependent acquisition based on a 1 s MS survey scan from 350 m/z to 1500 m/z, followed by up to 3 MS/MS scans of 2 s each. Single charged ions were excluded because in ESI mode, peptides typically carry multiple charges. Centroids were calculated for the acquired data that was converted to mzXML format using msConvert [49]. Peptides were identified from the human UniProtKB/SwissProt database containing canonical and isoform protein sequences (downloaded October 2013) using the search engine X!Tandem [2] in conjunction with PeptideProphet [11], both implemented in the Trans Proteomic Pipeline v4.3 [10], at an estimated false discovery rate (FDR) of 1%. Search parameters included a mass tolerance of 200 ppm for parental ions and 0.2 Da (Da) for fragment ions, allowing up to two missed cleavages. The following fixed peptide modifications were set: carbamidomethylation of cysteine side chains (+57.02 Da) and dimethylation of lysine Ɛ-amines (+28.03 Da); methionine oxidation (+15.99 Da). N-terminal dimethylation (+28.03 Da) and thioacylation (+88.00 Da) were set as variables. Note, N-terminally thioacylated peptides identified by LC–MS/MS represent prime-side cleavage products of the proteases of interest. The complete cleavage sites were reconstructed bioinformatically using the open web-based program WebPICS [12], available at http://clipserve.clip.ubc.ca/pics/, which generates a non-redundant list of identified cleavage sites by matching each prime side peptide sequence to the human IPI database (v3.69, 174784 entries; EMBL-EBI, UK) and extracting the non-prime cleavage side sequence up to the next cleavage site of the enzyme used for library generation, *i.e.* to the next N-terminal Asp or Glu for GluC-libraries, or the next N-terminal Arg or Lys in the case of trypsin-generated libraries. Subsite positions with ambiguous information coming *e.g.* from different protein isoforms are omitted and replaced by X for further analysis. Identified cleavage sites can be summarized as heat maps, by using *e.g.* Gnuplot (www.gnuplot.info), or iceLogos (http://iomics.ugent.be/icelogoserver/index.html) [29]

## 6 Quenched fluorescence protease activity assay

Synthetic quenched fluorescent (QF) peptides were purchased from ChinaPeptides Co. Ltd. (Shanghai, China), dissolved in DMSO and protected from light. Working stocks (100 μM) were prepared in DMSO using the molar extinction coefficient of the conjugated quencher (DNP; (2,4)-dinitrophenyl) of 6.985 cm^−1^mM^−1^ at 400 nm [50]. MMP zymogens (MMP1, MMP2, MMP8, MMP9, MMP14) were activated in 100 mM Tris, pH 7.5, 100 mM NaCl, 10 mM CaCl_2_, and 0.05% Brij-35, using 1 mM APMA (para-aminophenylmercuric acetate) at 37 °C for 30 min. Chymotrypsin was used to activate MMP3 at a ratio of 1:100 (w/w) for 30 min at 37 °C, and was subsequently inactivated using 1 mM PMSF. MMP7, MMP12, and MMP13 were typically auto-activated during purification. Quenched fluorescent peptide assays were performed immediately after MMP activation in the presence of protease inhibitor cocktail (HALT™, Life Technologies, no EDTA added) using a multi-wavelength fluorescence scanner (POLARstar OPTIMA, BMG Labtech). Each MMP (1–10 nM) was incubated with 1 µM QF-peptide in 100 µL of 100 mM Tris, pH 7.5, 100 mM NaCl, 10 mM CaCl_2_, and 0.05% Brij-35, and the increase in fluorescence was measured at 45 s intervals for 1 h at 37 °C. The excitation and emission wavelengths were set to 320 and 405 nm, respectively, and all measurements were performed in duplicate. Experiments were repeated three times with independent substrate and MMP preparations on consecutive days.
