# Supplementary material for: Active site specificity profiling datasets of matrix metalloproteinases (MMPs) 1, 2, 3, 7, 8, 9, 12, 13 and 14
Source: Data Brief. 2016 Feb 22;7:299–310. doi: 10.1016/j.dib.2016.02.036 (PMC4777984; doi:10.1016/j.dib.2016.02.036)
Supplement: Supplementary file 1 — Supplementary material [file mmc1.pdf]

Domain architectures of matrix metalloproteinases.

Supplementary Figure 1. (A) Minimal MMPs: MMPs 7 and 26 (matrilysin-1 and -2). **(B)** Classical MMPs such as MMPs 1, 3, 8, 12, and 13 possessing both catalytic domain (magenta) and hemopexin domain (dark green). **(C)** The gelatinases, MMPs 2 and 9, with inserted fibronectin-like type-II modules (olive) proximal to the active site. **(D)** Membrane-type MMPs with a C-terminal transmembrane (e.g. MMPs 14, 15, and 16) or GPI-anchor (MMPs 17 and 25; light gray). Signal- and pro-domains of zymogen forms are depicted in yellow and light green, respectively.

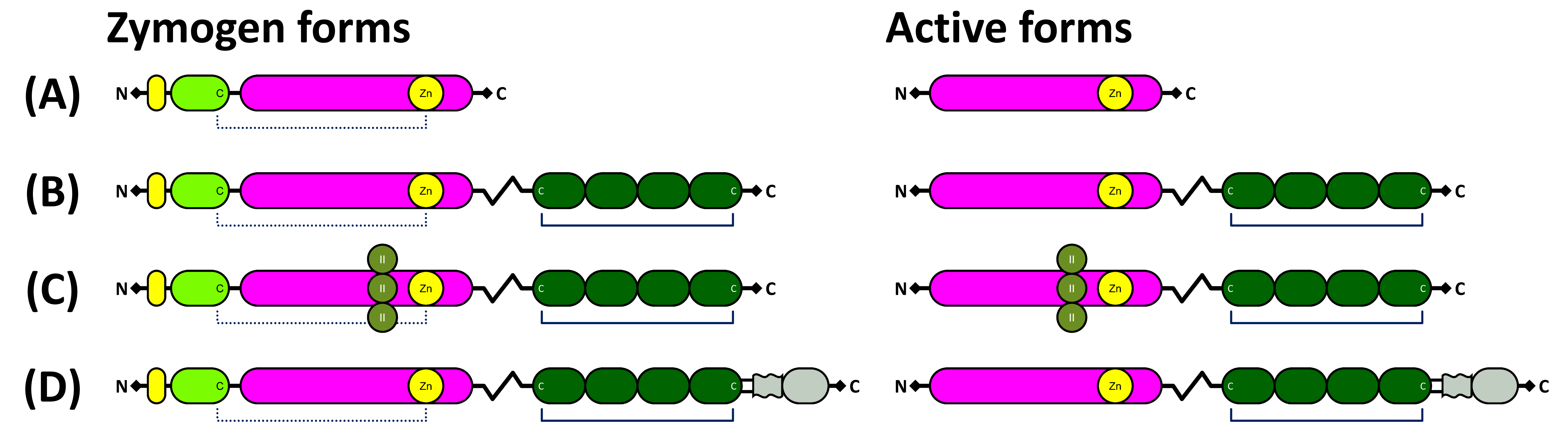

Eckhard et al. 2016

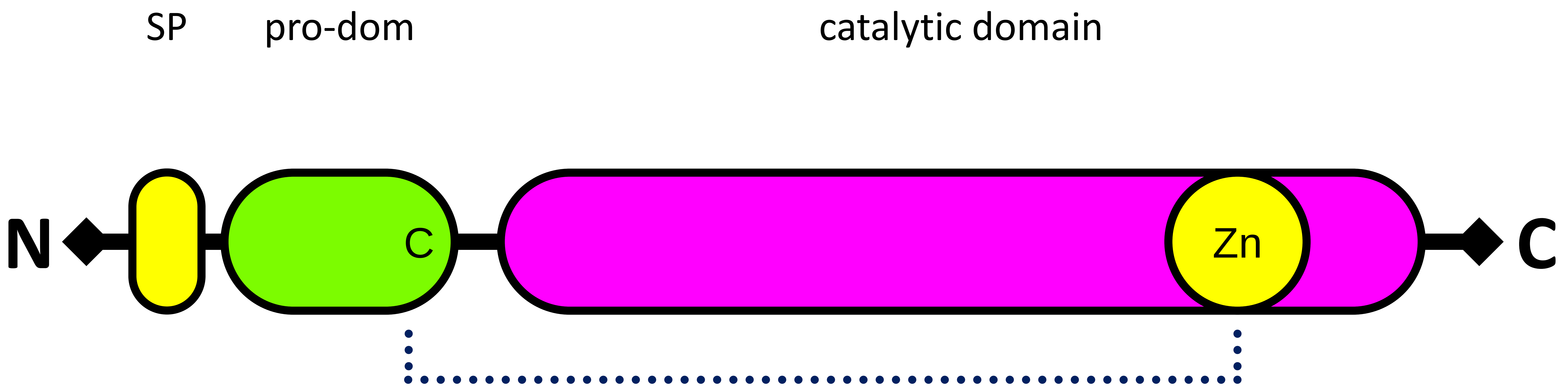

Eckhard et al. 2016

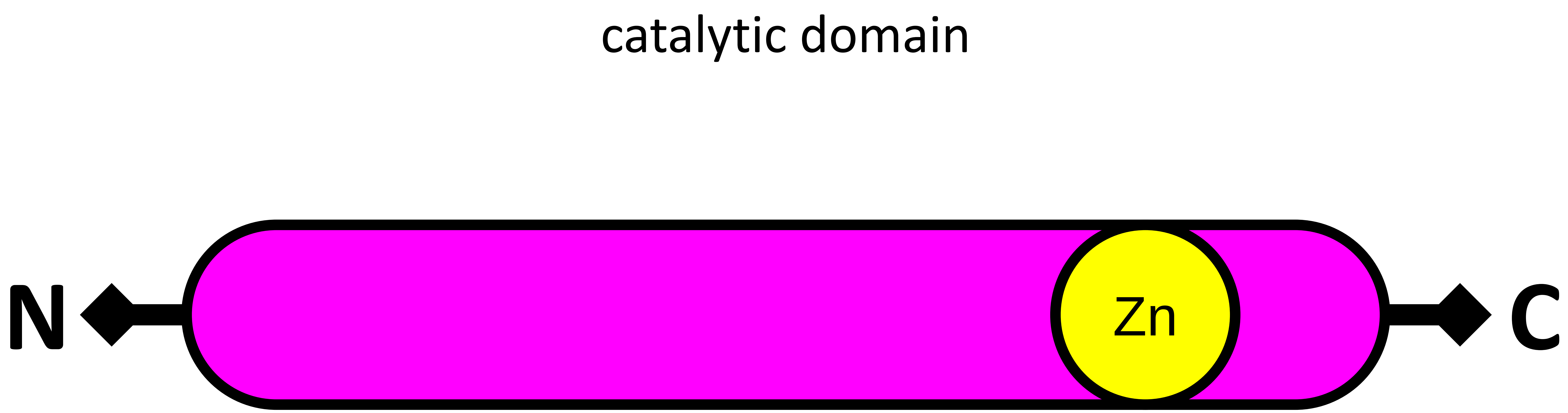

Eckhard et al. 2016

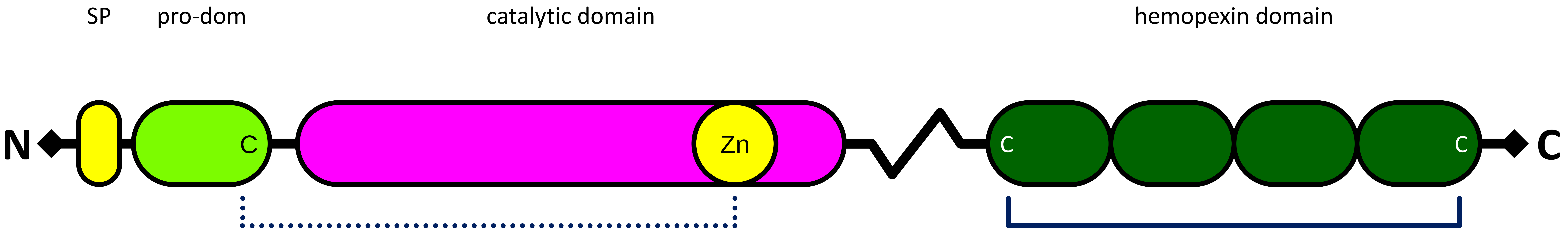

Eckhard et al. 2016

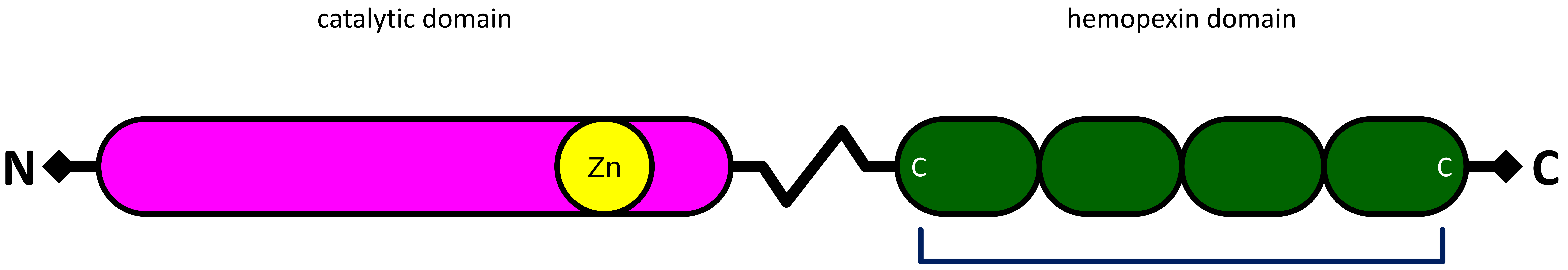

Eckhard et al. 2016

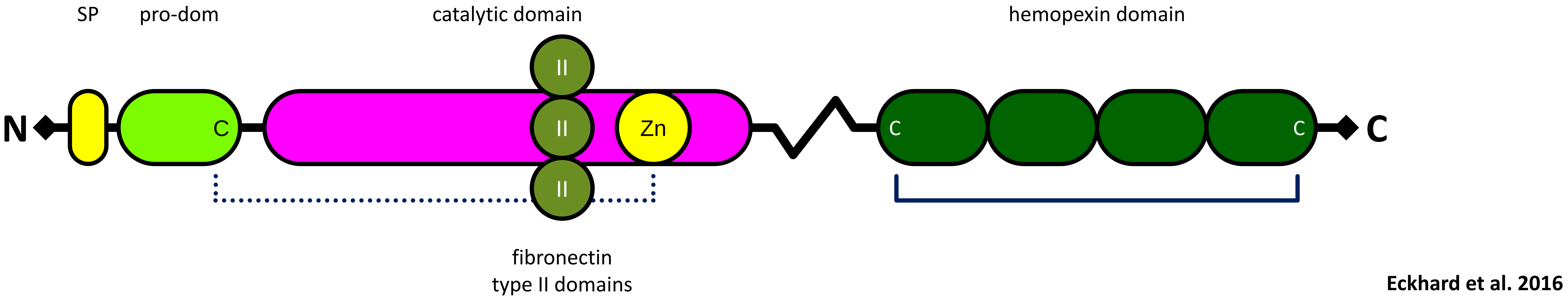
SP
pro-dom
catalytic domain
hemopexin domain
N
C
II
II
II
Zn
C
C
C
C
fibronectin
type II domains
Eckhard et al. 2016

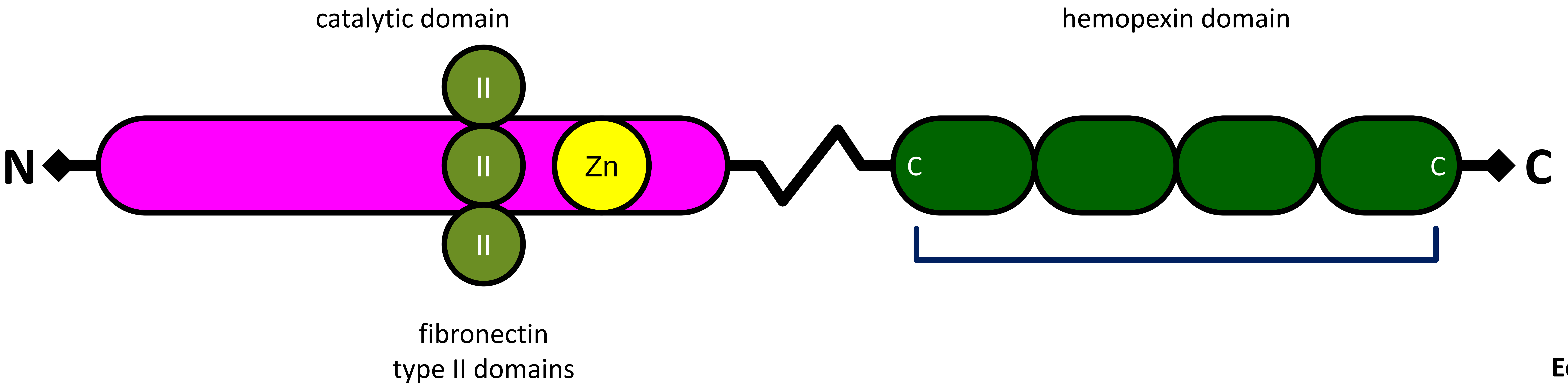

Eckhard et al. 2016

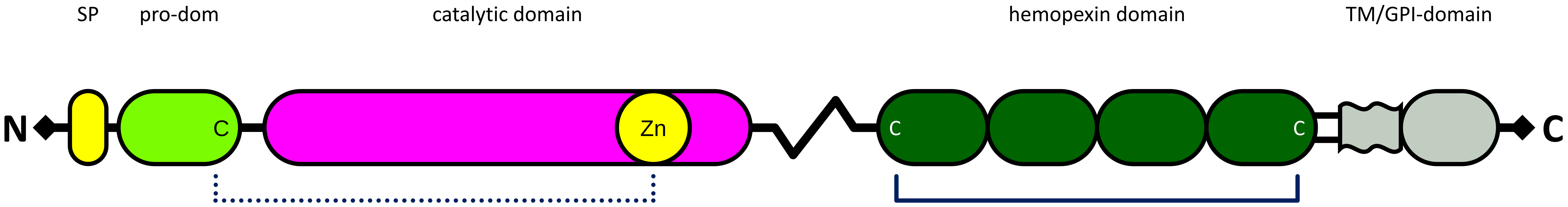

Eckhard et al. 2016

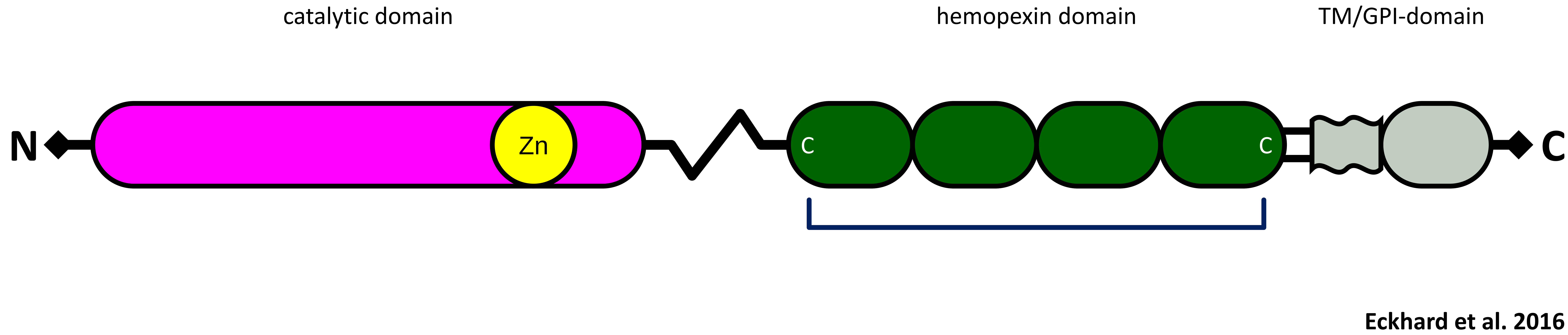
catalytic domain
hemopexin domain
TM/GPI-domain
N
Zn
C
C
C
Eckhard et al. 2016
