# Supplementary material for: Active site specificity profiling datasets of matrix metalloproteinases (MMPs) 1, 2, 3, 7, 8, 9, 12, 13 and 14
Source: Data Brief. 2016 Feb 22;7:299–310. doi: 10.1016/j.dib.2016.02.036 (PMC4777984; doi:10.1016/j.dib.2016.02.036)
Supplement: Supplementary file 3 — Supplementary material [file mmc3.pdf]

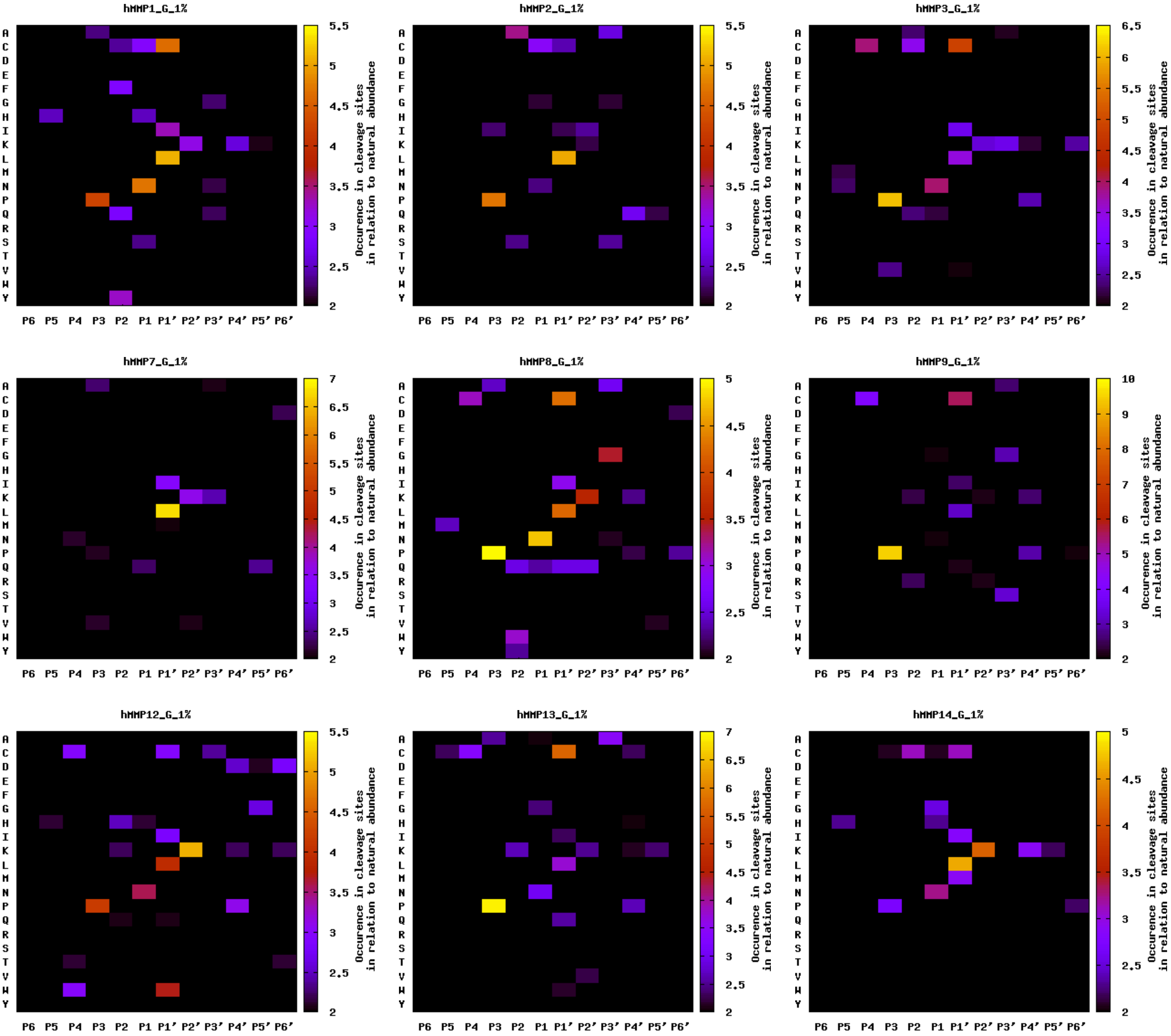

Supplementary Figure 3. Specificity profiles of MMPs 1, 2, 3, 7, 8, 9, 12, 13, and 14 using GluC-generated human peptide libraries. Identified cleavage sites are summarized as heat maps showing fold-change over natural abundance. P6 to P6′ subsite positions are shown on the x axes with the identified cleavage site between P1 and P1'. Plotted amino acids are indicated on the y axes with single-letter codes. Please refer to Supplementary Figure 3 for corresponding results using GluC-generated peptide libraries.
