# Supplementary material for: Active site specificity profiling datasets of matrix metalloproteinases (MMPs) 1, 2, 3, 7, 8, 9, 12, 13 and 14
Source: Data Brief. 2016 Feb 22;7:299–310. doi: 10.1016/j.dib.2016.02.036 (PMC4777984; doi:10.1016/j.dib.2016.02.036)
Supplement: Supplementary file 10 — Supplementary material [file mmc10.zip › WebPICS_hMMP12_G_1%/P1.html]

 

PICS results


|  |  |
| --- | --- |
| **P1\_A**  13 in 124 sites   10.5 %    effects > 10 perc. pnts.  (vice-versa in brackets)  P3\_V: 21.9 (25.9)   P2\_H: 17.5 (32.4)   P2\_K: 17.9 (14.5)   P1prime\_Q: 22.7 (29.5)   P2prime\_T: 21.1 (22.8)   P3prime\_A: 22.4 (14.5)   P3prime\_G: 15.8 (22.8)   P3prime\_K: 12.6 (12.6) |  |
  
| **P1\_D**  9 in 124 sites   7.3 %    effects > 10 perc. pnts.  (vice-versa in brackets)  P1prime\_C: 18.2 (32.7) |  |
  
| **P1\_G**  9 in 124 sites   7.3 %    effects > 10 perc. pnts.  (vice-versa in brackets)  P2\_A: 43.5 (26.0)   P1prime\_W: 29.3 (52.7)   P3prime\_C: 19.0 (42.7) |  |
  
| **P1\_H**  6 in 124 sites   4.8 %    effects > 10 perc. pnts.  (vice-versa in brackets)  P2\_Y: 13.5 (20.2)   P2prime\_Q: 12.7 (15.2) |  |
  
| **P1\_K**  10 in 124 sites   8.1 %    effects > 10 perc. pnts.  (vice-versa in brackets)  P2\_K: 27.1 (16.9)   P2\_N: 15.2 (25.2)   P1prime\_I: 33.1 (15.7)   P3prime\_D: 13.5 (16.9)   P3prime\_G: 12.7 (14.1) |  |
  
| **P1\_N**  18 in 124 sites   14.5 %    effects > 10 perc. pnts.  (vice-versa in brackets)  P3\_P: -13.8 (-10.3)   P1prime\_V: 19.6 (20.8)   P3prime\_A: 17.2 (15.5)   P3prime\_N: 14.1 (25.5) |  |
  
| **P1\_P**  10 in 124 sites   8.1 %    effects > 10 perc. pnts.  (vice-versa in brackets)  P2prime\_R: 12.7 (14.1)   P3prime\_V: 22.7 (25.2) |  |
  
| **P1\_Q**  9 in 124 sites   7.3 %    effects > 10 perc. pnts.  (vice-versa in brackets)  P3\_P: 36.2 (13.5)   P3\_V: 13.3 (10.9)   P2\_F: 37.9 (42.7)   P2\_Q: 14.1 (12.7)   P1prime\_V: 41.9 (22.1)   P2prime\_I: 45.1 (31.2)   P3prime\_N: 47.5 (42.7)   P3prime\_Q: 18.2 (32.7) |  |
  
| **P1\_S**  15 in 124 sites   12.1 %    effects > 10 perc. pnts.  (vice-versa in brackets)  P2\_Q: 11.9 (17.9)   P2prime\_V: 27.1 (25.4)   P3prime\_T: 12.7 (21.2) |  |
