# Supplementary material for: Active site specificity profiling datasets of matrix metalloproteinases (MMPs) 1, 2, 3, 7, 8, 9, 12, 13 and 14
Source: Data Brief. 2016 Feb 22;7:299–310. doi: 10.1016/j.dib.2016.02.036 (PMC4777984; doi:10.1016/j.dib.2016.02.036)
Supplement: Supplementary file 10 — Supplementary material [file mmc10.zip › WebPICS_hMMP12_G_1%/P1prime.html]

 

PICS results


|  |  |
| --- | --- |
| **P1prime\_C**  5 in 124 sites   4.0 %    effects > 10 perc. pnts.  (vice-versa in brackets)  P3\_A: 45.5 (12.7)   P2\_G: 28.7 (10.3)   P1\_D: 32.7 (18.2)   P2prime\_I: 29.5 (11.4)   P3prime\_C: 16.8 (21.0) |  |
  
| **P1prime\_I**  21 in 124 sites   16.9 %    effects > 10 perc. pnts.  (vice-versa in brackets)  P3\_V: 19.7 (37.6)   P1\_K: 15.7 (33.1)   P3prime\_A: 12.5 (13.1) |  |
  
| **P1prime\_Q**  10 in 124 sites   8.1 %    effects > 10 perc. pnts.  (vice-versa in brackets)  P3\_V: 11.1 (10.1)   P2\_K: 27.1 (16.9)   P1\_A: 29.5 (22.7)   P3prime\_G: 12.7 (14.1) |  |
  
| **P1prime\_V**  17 in 124 sites   13.7 %    effects > 10 perc. pnts.  (vice-versa in brackets)  P3\_P: 21.8 (15.5)   P2\_F: 28.8 (61.3)   P1\_N: 20.8 (19.6)   P1\_Q: 22.1 (41.9)   P2prime\_I: 30.7 (40.1)   P2prime\_V: 16.5 (17.5)   P3prime\_N: 33.1 (56.3) |  |
  
| **P1prime\_W**  5 in 124 sites   4.0 %    effects > 10 perc. pnts.  (vice-versa in brackets)  P2\_A: 47.9 (16.0)   P2\_H: 14.4 (10.3)   P1\_G: 52.7 (29.3)   P3prime\_K: 29.5 (11.4) |  |
