# Supplementary material for: Active site specificity profiling datasets of matrix metalloproteinases (MMPs) 1, 2, 3, 7, 8, 9, 12, 13 and 14
Source: Data Brief. 2016 Feb 22;7:299–310. doi: 10.1016/j.dib.2016.02.036 (PMC4777984; doi:10.1016/j.dib.2016.02.036)
Supplement: Supplementary file 10 — Supplementary material [file mmc10.zip › WebPICS_hMMP12_G_1%/P2.html]

 

PICS results


|  |  |
| --- | --- |
| **P2\_A**  15 in 124 sites   12.1 %    effects > 10 perc. pnts.  (vice-versa in brackets)  P3\_P: 20.6 (12.9)   P1\_G: 26.0 (43.5)   P1prime\_W: 16.0 (47.9)   P2prime\_K: 30.2 (12.2) |  |
  
| **P2\_C**  2 in 124 sites   1.6 %    effects > 10 perc. pnts.  (vice-versa in brackets)  P2prime\_H: 96.8 (48.4) |  |
  
| **P2\_F**  8 in 124 sites   6.5 %    effects > 10 perc. pnts.  (vice-versa in brackets)  P3\_A: 23.0 (10.2)   P3\_P: 30.6 (10.2)   P1\_Q: 42.7 (37.9)   P1prime\_V: 61.3 (28.8)   P2prime\_I: 64.5 (39.7)   P3prime\_D: 18.5 (18.5)   P3prime\_N: 66.9 (53.5) |  |
  
| **P2\_G**  14 in 124 sites   11.3 %    effects > 10 perc. pnts.  (vice-versa in brackets)  P1prime\_C: 10.3 (28.7) |  |
  
| **P2\_H**  7 in 124 sites   5.6 %    effects > 10 perc. pnts.  (vice-versa in brackets)  P3\_V: 34.0 (21.7)   P1\_A: 32.4 (17.5)   P1prime\_W: 10.3 (14.4)   P2prime\_T: 33.2 (19.4) |  |
  
| **P2\_K**  16 in 124 sites   12.9 %    effects > 10 perc. pnts.  (vice-versa in brackets)  P3\_P: 30.6 (20.4)   P1\_A: 14.5 (17.9)   P1\_K: 16.9 (27.1)   P1prime\_Q: 16.9 (27.1)   P2prime\_V: 18.3 (18.3)   P3prime\_D: 12.3 (24.6)   P3prime\_G: 11.5 (20.4) |  |
  
| **P2\_N**  6 in 124 sites   4.8 %    effects > 10 perc. pnts.  (vice-versa in brackets)  P1\_K: 25.2 (15.2)   P3prime\_T: 26.0 (17.4) |  |
  
| **P2\_Q**  10 in 124 sites   8.1 %    effects > 10 perc. pnts.  (vice-versa in brackets)  P1\_Q: 12.7 (14.1)   P1\_S: 17.9 (11.9)   P2prime\_I: 19.5 (15.0)   P3prime\_C: 16.8 (41.9)   P3prime\_Q: 16.0 (31.9) |  |
  
| **P2\_Y**  4 in 124 sites   3.2 %    effects > 10 perc. pnts.  (vice-versa in brackets)  P1\_H: 20.2 (13.5)   P2prime\_Q: 21.0 (16.8)   P3prime\_R: 19.4 (11.1) |  |
