# Supplementary material for: Active site specificity profiling datasets of matrix metalloproteinases (MMPs) 1, 2, 3, 7, 8, 9, 12, 13 and 14
Source: Data Brief. 2016 Feb 22;7:299–310. doi: 10.1016/j.dib.2016.02.036 (PMC4777984; doi:10.1016/j.dib.2016.02.036)
Supplement: Supplementary file 10 — Supplementary material [file mmc10.zip › WebPICS_hMMP12_G_1%/P2prime.html]

 

PICS results


|  |  |
| --- | --- |
| **P2prime\_H**  4 in 124 sites   3.2 %    effects > 10 perc. pnts.  (vice-versa in brackets)  P2\_C: 48.4 (96.8) |  |
  
| **P2prime\_I**  13 in 124 sites   10.5 %    effects > 10 perc. pnts.  (vice-versa in brackets)  P3\_A: 24.0 (17.3)   P3\_P: 19.1 (10.3)   P2\_F: 39.7 (64.5)   P2\_Q: 15.0 (19.5)   P1\_Q: 31.2 (45.1)   P1prime\_C: 11.4 (29.5)   P1prime\_V: 40.1 (30.7)   P3prime\_N: 38.1 (49.5) |  |
  
| **P2prime\_K**  37 in 124 sites   29.8 %    effects > 10 perc. pnts.  (vice-versa in brackets)  P2\_A: 12.2 (30.2) |  |
  
| **P2prime\_Q**  5 in 124 sites   4.0 %    effects > 10 perc. pnts.  (vice-versa in brackets)  P3\_H: 17.6 (29.3)   P2\_Y: 16.8 (21.0)   P1\_H: 15.2 (12.7) |  |
  
| **P2prime\_R**  9 in 124 sites   7.3 %    effects > 10 perc. pnts.  (vice-versa in brackets)  P1\_P: 14.1 (12.7) |  |
  
| **P2prime\_T**  12 in 124 sites   9.7 %    effects > 10 perc. pnts.  (vice-versa in brackets)  P3\_V: 41.1 (44.8)   P2\_H: 19.4 (33.2)   P1\_A: 22.8 (21.1)   P3prime\_A: 33.9 (20.3) |  |
  
| **P2prime\_V**  16 in 124 sites   12.9 %    effects > 10 perc. pnts.  (vice-versa in brackets)  P2\_K: 18.3 (18.3)   P1\_S: 25.4 (27.1)   P1prime\_V: 17.5 (16.5) |  |
