# Supplementary material for: Active site specificity profiling datasets of matrix metalloproteinases (MMPs) 1, 2, 3, 7, 8, 9, 12, 13 and 14
Source: Data Brief. 2016 Feb 22;7:299–310. doi: 10.1016/j.dib.2016.02.036 (PMC4777984; doi:10.1016/j.dib.2016.02.036)
Supplement: Supplementary file 10 — Supplementary material [file mmc10.zip › WebPICS_hMMP12_G_1%/P3.html]

 

PICS results


|  |  |
| --- | --- |
| **P3\_A**  18 in 124 sites   14.5 %    effects > 10 perc. pnts.  (vice-versa in brackets)  P1prime\_C: 12.7 (45.5)   P2\_F: 10.2 (23.0)   P2prime\_I: 17.3 (24.0)   P3prime\_K: 11.7 (16.3) |  |
  
| **P3\_H**  3 in 124 sites   2.4 %    effects > 10 perc. pnts.  (vice-versa in brackets)  P2prime\_Q: 29.3 (17.6)   P3prime\_V: 92.7 (30.9) |  |
  
| **P3\_P**  24 in 124 sites   19.4 %    effects > 10 perc. pnts.  (vice-versa in brackets)  P1\_N: -10.3 (-13.8)   P1\_Q: 13.5 (36.2)   P1prime\_V: 15.5 (21.8)   P2\_A: 12.9 (20.6)   P2\_F: 10.2 (30.6)   P2\_K: 20.4 (30.6)   P2prime\_I: 10.3 (19.1) |  |
  
| **P3\_V**  11 in 124 sites   8.9 %    effects > 10 perc. pnts.  (vice-versa in brackets)  P1\_A: 25.9 (21.9)   P1\_Q: 10.9 (13.3)   P1prime\_I: 37.6 (19.7)   P1prime\_Q: 10.1 (11.1)   P2\_H: 21.7 (34.0)   P2prime\_T: 44.8 (41.1)   P3prime\_A: 20.3 (11.1) |  |
