# Supplementary material for: Active site specificity profiling datasets of matrix metalloproteinases (MMPs) 1, 2, 3, 7, 8, 9, 12, 13 and 14
Source: Data Brief. 2016 Feb 22;7:299–310. doi: 10.1016/j.dib.2016.02.036 (PMC4777984; doi:10.1016/j.dib.2016.02.036)
Supplement: Supplementary file 10 — Supplementary material [file mmc10.zip › WebPICS_hMMP12_G_1%/P3prime.html]

 

PICS results


|  |  |
| --- | --- |
| **P3prime\_A**  20 in 124 sites   16.1 %    effects > 10 perc. pnts.   (vice-versa in brackets)  P3\_V: 11.1 (20.3)   P1\_A: 14.5 (22.4)   P1\_N: 15.5 (17.2)   P1prime\_I: 13.1 (12.5)   P2prime\_T: 20.3 (33.9) |  |
  
| **P3prime\_C**  4 in 124 sites   3.2 %    effects > 10 perc. pnts.   (vice-versa in brackets)  P2\_Q: 41.9 (16.8)   P1\_G: 42.7 (19.0)   P1prime\_C: 21.0 (16.8) |  |
  
| **P3prime\_D**  8 in 124 sites   6.5 %    effects > 10 perc. pnts.   (vice-versa in brackets)  P2\_F: 18.5 (18.5)   P2\_K: 24.6 (12.3)   P1\_K: 16.9 (13.5) |  |
  
| **P3prime\_G**  9 in 124 sites   7.3 %    effects > 10 perc. pnts.   (vice-versa in brackets)  P2\_K: 20.4 (11.5)   P1\_A: 22.8 (15.8)   P1\_K: 14.1 (12.7)   P1prime\_Q: 14.1 (12.7) |  |
  
| **P3prime\_K**  13 in 124 sites   10.5 %    effects > 10 perc. pnts.   (vice-versa in brackets)  P3\_A: 16.3 (11.7)   P1\_A: 12.6 (12.6)   P1prime\_W: 11.4 (29.5) |  |
  
| **P3prime\_N**  10 in 124 sites   8.1 %    effects > 10 perc. pnts.   (vice-versa in brackets)  P2\_F: 53.5 (66.9)   P1\_N: 25.5 (14.1)   P1\_Q: 42.7 (47.5)   P1prime\_V: 56.3 (33.1)   P2prime\_I: 49.5 (38.1) |  |
  
| **P3prime\_Q**  5 in 124 sites   4.0 %    effects > 10 perc. pnts.   (vice-versa in brackets)  P2\_Q: 31.9 (16.0)   P1\_Q: 32.7 (18.2) |  |
  
| **P3prime\_R**  7 in 124 sites   5.6 %    effects > 10 perc. pnts.   (vice-versa in brackets)  P2\_Y: 11.1 (19.4) |  |
  
| **P3prime\_T**  9 in 124 sites   7.3 %    effects > 10 perc. pnts.   (vice-versa in brackets)  P2\_N: 17.4 (26.0)   P1\_S: 21.2 (12.7) |  |
  
| **P3prime\_V**  9 in 124 sites   7.3 %    effects > 10 perc. pnts.   (vice-versa in brackets)  P3\_H: 30.9 (92.7)   P1\_P: 25.2 (22.7) |  |
