# Supplementary material for: Active site specificity profiling datasets of matrix metalloproteinases (MMPs) 1, 2, 3, 7, 8, 9, 12, 13 and 14
Source: Data Brief. 2016 Feb 22;7:299–310. doi: 10.1016/j.dib.2016.02.036 (PMC4777984; doi:10.1016/j.dib.2016.02.036)
Supplement: Supplementary file 10 — Supplementary material [file mmc10.zip › WebPICS_hMMP12_G_1%/subsite_coop.html]

 

PICS results


- # About
- # Manual
- # Analysis
- # Results

- Redraw
- Seqlogo
- Dependency
- Coop

**Potential subsite cooperativity analysis for hMMP12\_G\_1%**   
Minimum difference for subsite dependency was set to +- 10 percentage-points  
Subsite dependency was checked for all positional occurences above 1 x natural abundance  
**Subsite dependency check is restricted to P3 - P3'**

|  |  |  |  |
| --- | --- | --- | --- |
| Fixed residue | Affected residue(s) | Change (percentage-points) | Vice-Versa change |
| P3\_A | P1prime\_C | 12.7 | 45.5 |
| P3\_A | P2\_F | 10.2 | 23.0 |
| P3\_A | P2prime\_I | 17.3 | 24.0 |
| P3\_A | P3prime\_K | 11.7 | 16.3 |
| P3\_H | P2prime\_Q | 29.3 | 17.6 |
| P3\_H | P3prime\_V | 92.7 | 30.9 |
| P3\_P | P1\_N | -10.3 | -13.8 |
| P3\_P | P1\_Q | 13.5 | 36.2 |
| P3\_P | P1prime\_V | 15.5 | 21.8 |
| P3\_P | P2\_A | 12.9 | 20.6 |
| P3\_P | P2\_F | 10.2 | 30.6 |
| P3\_P | P2\_K | 20.4 | 30.6 |
| P3\_P | P2prime\_I | 10.3 | 19.1 |
| P3\_V | P1\_A | 25.9 | 21.9 |
| P3\_V | P1\_Q | 10.9 | 13.3 |
| P3\_V | P1prime\_I | 37.6 | 19.7 |
| P3\_V | P1prime\_Q | 10.1 | 11.1 |
| P3\_V | P2\_H | 21.7 | 34.0 |
| P3\_V | P2prime\_T | 44.8 | 41.1 |
| P3\_V | P3prime\_A | 20.3 | 11.1 |
| P2\_A | P1\_G | 26.0 | 43.5 |
| P2\_A | P1prime\_W | 16.0 | 47.9 |
| P2\_A | P2prime\_K | 30.2 | 12.2 |
| P2\_C | P2prime\_H | 96.8 | 48.4 |
| P2\_F | P1\_Q | 42.7 | 37.9 |
| P2\_F | P1prime\_V | 61.3 | 28.8 |
| P2\_F | P2prime\_I | 64.5 | 39.7 |
| P2\_F | P3prime\_D | 18.5 | 18.5 |
| P2\_F | P3prime\_N | 66.9 | 53.5 |
| P2\_G | P1prime\_C | 10.3 | 28.7 |
| P2\_H | P1\_A | 32.4 | 17.5 |
| P2\_H | P1prime\_W | 10.3 | 14.4 |
| P2\_H | P2prime\_T | 33.2 | 19.4 |
| P2\_K | P1\_A | 14.5 | 17.9 |
| P2\_K | P1\_K | 16.9 | 27.1 |
| P2\_K | P1prime\_Q | 16.9 | 27.1 |
| P2\_K | P2prime\_V | 18.3 | 18.3 |
| P2\_K | P3prime\_D | 12.3 | 24.6 |
| P2\_K | P3prime\_G | 11.5 | 20.4 |
| P2\_N | P1\_K | 25.2 | 15.2 |
| P2\_N | P3prime\_T | 26.0 | 17.4 |
| P2\_Q | P1\_Q | 12.7 | 14.1 |
| P2\_Q | P1\_S | 17.9 | 11.9 |
| P2\_Q | P2prime\_I | 19.5 | 15.0 |
| P2\_Q | P3prime\_C | 16.8 | 41.9 |
| P2\_Q | P3prime\_Q | 16.0 | 31.9 |
| P2\_Y | P1\_H | 20.2 | 13.5 |
| P2\_Y | P2prime\_Q | 21.0 | 16.8 |
| P2\_Y | P3prime\_R | 19.4 | 11.1 |
| P1\_A | P1prime\_Q | 22.7 | 29.5 |
| P1\_A | P2prime\_T | 21.1 | 22.8 |
| P1\_A | P3prime\_A | 22.4 | 14.5 |
| P1\_A | P3prime\_G | 15.8 | 22.8 |
| P1\_A | P3prime\_K | 12.6 | 12.6 |
| P1\_D | P1prime\_C | 18.2 | 32.7 |
| P1\_G | P1prime\_W | 29.3 | 52.7 |
| P1\_G | P3prime\_C | 19.0 | 42.7 |
| P1\_H | P2prime\_Q | 12.7 | 15.2 |
| P1\_K | P1prime\_I | 33.1 | 15.7 |
| P1\_K | P3prime\_D | 13.5 | 16.9 |
| P1\_K | P3prime\_G | 12.7 | 14.1 |
| P1\_N | P1prime\_V | 19.6 | 20.8 |
| P1\_N | P3prime\_A | 17.2 | 15.5 |
| P1\_N | P3prime\_N | 14.1 | 25.5 |
| P1\_P | P2prime\_R | 12.7 | 14.1 |
| P1\_P | P3prime\_V | 22.7 | 25.2 |
| P1\_Q | P1prime\_V | 41.9 | 22.1 |
| P1\_Q | P2prime\_I | 45.1 | 31.2 |
| P1\_Q | P3prime\_N | 47.5 | 42.7 |
| P1\_Q | P3prime\_Q | 18.2 | 32.7 |
| P1\_S | P2prime\_V | 27.1 | 25.4 |
| P1\_S | P3prime\_T | 12.7 | 21.2 |
| P1prime\_C | P2prime\_I | 29.5 | 11.4 |
| P1prime\_C | P3prime\_C | 16.8 | 21.0 |
| P1prime\_I | P3prime\_A | 12.5 | 13.1 |
| P1prime\_Q | P3prime\_G | 12.7 | 14.1 |
| P1prime\_V | P2prime\_I | 30.7 | 40.1 |
| P1prime\_V | P2prime\_V | 16.5 | 17.5 |
| P1prime\_V | P3prime\_N | 33.1 | 56.3 |
| P1prime\_W | P3prime\_K | 29.5 | 11.4 |
| P2prime\_I | P3prime\_N | 38.1 | 49.5 |
| P2prime\_T | P3prime\_A | 33.9 | 20.3 |

  
**PICS analysis of protease: hMMP12\_G\_1%**  
124 cleavage sites analyzed  
PICS library made with (T)rypsin, (G)luC or (C)hymotrypsin:   
Cutoff for graphic display: 2 x natural abundance  

|  |  |
| --- | --- |
| Positional occurences  (table for total and relative (in %) values) | Occurences relative to natural abundance (table) |
|  |  |
