# Supplementary material for: Active site specificity profiling datasets of matrix metalloproteinases (MMPs) 1, 2, 3, 7, 8, 9, 12, 13 and 14
Source: Data Brief. 2016 Feb 22;7:299–310. doi: 10.1016/j.dib.2016.02.036 (PMC4777984; doi:10.1016/j.dib.2016.02.036)
Supplement: Supplementary file 10 — Supplementary material [file mmc10.zip › WebPICS_hMMP12_T_1%/P1.html]

 

PICS results


|  |  |
| --- | --- |
| **P1\_D**  32 in 275 sites   11.6 %    effects > 10 perc. pnts.  (vice-versa in brackets)  P2\_L: 13.0 (12.6)   P1prime\_Y: 18.1 (30.5) |  |
  
| **P1\_E**  29 in 275 sites   10.5 %    effects > 10 perc. pnts.  (vice-versa in brackets)  P2prime\_Q: 10.3 (15.8) |  |
  
| **P1\_N**  39 in 275 sites   14.2 %    effects > 10 perc. pnts.  (vice-versa in brackets)  P2\_G: 12.9 (17.9) |  |
  
| **P1\_Q**  16 in 275 sites   5.8 %    effects > 10 perc. pnts.  (vice-versa in brackets)  P3\_C: 17.3 (69.2)   P2\_D: 12.6 (11.8)   P2\_N: 13.7 (15.6)   P1prime\_C: 16.3 (37.1)   P2prime\_I: 16.6 (11.6) |  |
  
| **P1\_S**  24 in 275 sites   8.7 %    effects > 10 perc. pnts.  (vice-versa in brackets)  P3\_A: 20.8 (10.9)   P3prime\_G: 16.5 (11.3) |  |
