# Supplementary material for: Active site specificity profiling datasets of matrix metalloproteinases (MMPs) 1, 2, 3, 7, 8, 9, 12, 13 and 14
Source: Data Brief. 2016 Feb 22;7:299–310. doi: 10.1016/j.dib.2016.02.036 (PMC4777984; doi:10.1016/j.dib.2016.02.036)
Supplement: Supplementary file 10 — Supplementary material [file mmc10.zip › WebPICS_hMMP12_T_1%/P1prime.html]

 

PICS results


|  |  |
| --- | --- |
| **P1prime\_C**  7 in 275 sites   2.5 %    effects > 10 perc. pnts.  (vice-versa in brackets)  P3\_C: 41.4 (72.5)   P2\_N: 37.8 (18.9)   P1\_Q: 37.1 (16.3) |  |
  
| **P1prime\_L**  79 in 275 sites   28.7 %    effects > 10 perc. pnts.  (vice-versa in brackets)  P2\_E: 11.2 (27.5) |  |
  
| **P1prime\_Q**  23 in 275 sites   8.4 %    effects > 10 perc. pnts.  (vice-versa in brackets)  P2prime\_Q: 14.8 (17.9) |  |
  
| **P1prime\_V**  36 in 275 sites   13.1 %    effects > 10 perc. pnts.  (vice-versa in brackets)  P2\_A: 11.9 (11.9)   P2prime\_T: 19.4 (30.4) |  |
  
| **P1prime\_Y**  19 in 275 sites   6.9 %    effects > 10 perc. pnts.  (vice-versa in brackets)  P2\_N: 10.7 (14.5)   P1\_D: 30.5 (18.1) |  |
