# Supplementary material for: Active site specificity profiling datasets of matrix metalloproteinases (MMPs) 1, 2, 3, 7, 8, 9, 12, 13 and 14
Source: Data Brief. 2016 Feb 22;7:299–310. doi: 10.1016/j.dib.2016.02.036 (PMC4777984; doi:10.1016/j.dib.2016.02.036)
Supplement: Supplementary file 10 — Supplementary material [file mmc10.zip › WebPICS_hMMP12_T_1%/P2.html]

 

PICS results


|  |  |
| --- | --- |
| **P2\_A**  36 in 275 sites   13.1 %    effects > 10 perc. pnts.  (vice-versa in brackets)  P3\_A: 13.9 (10.8)   P1prime\_V: 11.9 (11.9)   P2prime\_V: -11.4 (-10.5) |  |
  
| **P2\_D**  17 in 275 sites   6.2 %    effects > 10 perc. pnts.  (vice-versa in brackets)  P1\_Q: 11.8 (12.6) |  |
  
| **P2\_E**  32 in 275 sites   11.6 %    effects > 10 perc. pnts.  (vice-versa in brackets)  P1prime\_L: 27.5 (11.2)   P2prime\_K: 10.3 (10.3) |  |
  
| **P2\_F**  12 in 275 sites   4.4 %    effects > 10 perc. pnts.  (vice-versa in brackets)  P2prime\_I: 24.9 (13.0) |  |
  
| **P2\_G**  28 in 275 sites   10.2 %    effects > 10 perc. pnts.  (vice-versa in brackets)  P1\_N: 17.9 (12.9) |  |
  
| **P2\_L**  33 in 275 sites   12.0 %    effects > 10 perc. pnts.  (vice-versa in brackets)  P1\_D: 12.6 (13.0) |  |
  
| **P2\_N**  14 in 275 sites   5.1 %    effects > 10 perc. pnts.  (vice-versa in brackets)  P3\_C: 19.9 (69.9)   P1\_Q: 15.6 (13.7)   P1prime\_C: 18.9 (37.8)   P1prime\_Y: 14.5 (10.7)   P2prime\_K: 24.1 (10.5)   P3prime\_E: 25.5 (12.8) |  |
  
| **P2\_Q**  14 in 275 sites   5.1 %    effects > 10 perc. pnts.  (vice-versa in brackets)  P2prime\_Q: 21.7 (16.0) |  |
  
| **P2\_S**  21 in 275 sites   7.6 %    effects > 10 perc. pnts.  (vice-versa in brackets)  P3\_I: 15.1 (13.2)   P2prime\_H: 12.5 (14.6)   P3prime\_G: 20.6 (12.4) |  |
