# Supplementary material for: Active site specificity profiling datasets of matrix metalloproteinases (MMPs) 1, 2, 3, 7, 8, 9, 12, 13 and 14
Source: Data Brief. 2016 Feb 22;7:299–310. doi: 10.1016/j.dib.2016.02.036 (PMC4777984; doi:10.1016/j.dib.2016.02.036)
Supplement: Supplementary file 10 — Supplementary material [file mmc10.zip › WebPICS_hMMP12_T_1%/P2prime.html]

 

PICS results


|  |  |
| --- | --- |
| **P2prime\_H**  18 in 275 sites   6.5 %    effects > 10 perc. pnts.  (vice-versa in brackets)  P3\_I: 13.5 (10.2)   P2\_S: 14.6 (12.5) |  |
  
| **P2prime\_I**  23 in 275 sites   8.4 %    effects > 10 perc. pnts.  (vice-versa in brackets)  P2\_F: 13.0 (24.9)   P1\_Q: 11.6 (16.6)   P3prime\_S: 13.3 (13.3) |  |
  
| **P2prime\_K**  32 in 275 sites   11.6 %    effects > 10 perc. pnts.  (vice-versa in brackets)  P2\_E: 10.3 (10.3)   P2\_N: 10.5 (24.1)   P3prime\_E: 21.0 (24.1) |  |
  
| **P2prime\_Q**  19 in 275 sites   6.9 %    effects > 10 perc. pnts.  (vice-versa in brackets)  P2\_Q: 16.0 (21.7)   P1\_E: 15.8 (10.3)   P1prime\_Q: 17.9 (14.8) |  |
  
| **P2prime\_T**  23 in 275 sites   8.4 %    effects > 10 perc. pnts.  (vice-versa in brackets)  P3\_I: 13.0 (12.4)   P1prime\_V: 30.4 (19.4) |  |
  
| **P2prime\_V**  39 in 275 sites   14.2 %    effects > 10 perc. pnts.  (vice-versa in brackets)  P2\_A: -10.5 (-11.4)   P3prime\_T: 10.7 (15.4) |  |
