# Supplementary material for: Active site specificity profiling datasets of matrix metalloproteinases (MMPs) 1, 2, 3, 7, 8, 9, 12, 13 and 14
Source: Data Brief. 2016 Feb 22;7:299–310. doi: 10.1016/j.dib.2016.02.036 (PMC4777984; doi:10.1016/j.dib.2016.02.036)
Supplement: Supplementary file 10 — Supplementary material [file mmc10.zip › WebPICS_hMMP12_T_1%/P3.html]

 

PICS results


|  |  |
| --- | --- |
| **P3\_A**  46 in 275 sites   16.7 %    effects > 10 perc. pnts.  (vice-versa in brackets)  P1\_S: 10.9 (20.8)   P2\_A: 10.8 (13.9) |  |
  
| **P3\_C**  4 in 275 sites   1.5 %    effects > 10 perc. pnts.  (vice-versa in brackets)  P1\_Q: 69.2 (17.3)   P1prime\_C: 72.5 (41.4)   P2\_N: 69.9 (19.9) |  |
  
| **P3\_I**  24 in 275 sites   8.7 %    effects > 10 perc. pnts.  (vice-versa in brackets)  P2\_S: 13.2 (15.1)   P2prime\_H: 10.2 (13.5)   P2prime\_T: 12.4 (13.0)   P3prime\_D: 14.8 (12.7) |  |
