# Supplementary material for: Active site specificity profiling datasets of matrix metalloproteinases (MMPs) 1, 2, 3, 7, 8, 9, 12, 13 and 14
Source: Data Brief. 2016 Feb 22;7:299–310. doi: 10.1016/j.dib.2016.02.036 (PMC4777984; doi:10.1016/j.dib.2016.02.036)
Supplement: Supplementary file 10 — Supplementary material [file mmc10.zip › WebPICS_hMMP12_T_1%/P3prime.html]

 

PICS results


|  |  |
| --- | --- |
| **P3prime\_D**  28 in 275 sites   10.2 %    effects > 10 perc. pnts.   (vice-versa in brackets)  P3\_I: 12.7 (14.8) |  |
  
| **P3prime\_E**  28 in 275 sites   10.2 %    effects > 10 perc. pnts.   (vice-versa in brackets)  P2\_N: 12.8 (25.5)   P2prime\_K: 24.1 (21.0) |  |
  
| **P3prime\_G**  35 in 275 sites   12.7 %    effects > 10 perc. pnts.   (vice-versa in brackets)  P2\_S: 12.4 (20.6)   P1\_S: 11.3 (16.5) |  |
  
| **P3prime\_S**  23 in 275 sites   8.4 %    effects > 10 perc. pnts.   (vice-versa in brackets)  P2prime\_I: 13.3 (13.3) |  |
  
| **P3prime\_T**  27 in 275 sites   9.8 %    effects > 10 perc. pnts.   (vice-versa in brackets)  P2prime\_V: 15.4 (10.7) |  |
