# Supplementary material for: Active site specificity profiling datasets of matrix metalloproteinases (MMPs) 1, 2, 3, 7, 8, 9, 12, 13 and 14
Source: Data Brief. 2016 Feb 22;7:299–310. doi: 10.1016/j.dib.2016.02.036 (PMC4777984; doi:10.1016/j.dib.2016.02.036)
Supplement: Supplementary file 10 — Supplementary material [file mmc10.zip › WebPICS_hMMP12_T_1%/subsite_coop.html]

 

PICS results


- # About
- # Manual
- # Analysis
- # Results

- Redraw
- Seqlogo
- Dependency
- Coop

**Potential subsite cooperativity analysis for hMMP12\_T\_1%**   
Minimum difference for subsite dependency was set to +- 10 percentage-points  
Subsite dependency was checked for all positional occurences above 1 x natural abundance  
**Subsite dependency check is restricted to P3 - P3'**

|  |  |  |  |
| --- | --- | --- | --- |
| Fixed residue | Affected residue(s) | Change (percentage-points) | Vice-Versa change |
| P3\_A | P1\_S | 10.9 | 20.8 |
| P3\_A | P2\_A | 10.8 | 13.9 |
| P3\_C | P1\_Q | 69.2 | 17.3 |
| P3\_C | P1prime\_C | 72.5 | 41.4 |
| P3\_C | P2\_N | 69.9 | 19.9 |
| P3\_I | P2\_S | 13.2 | 15.1 |
| P3\_I | P2prime\_H | 10.2 | 13.5 |
| P3\_I | P2prime\_T | 12.4 | 13.0 |
| P3\_I | P3prime\_D | 14.8 | 12.7 |
| P2\_A | P1prime\_V | 11.9 | 11.9 |
| P2\_A | P2prime\_V | -11.4 | -10.5 |
| P2\_D | P1\_Q | 11.8 | 12.6 |
| P2\_E | P1prime\_L | 27.5 | 11.2 |
| P2\_E | P2prime\_K | 10.3 | 10.3 |
| P2\_F | P2prime\_I | 24.9 | 13.0 |
| P2\_G | P1\_N | 17.9 | 12.9 |
| P2\_L | P1\_D | 12.6 | 13.0 |
| P2\_N | P1\_Q | 15.6 | 13.7 |
| P2\_N | P1prime\_C | 18.9 | 37.8 |
| P2\_N | P1prime\_Y | 14.5 | 10.7 |
| P2\_N | P2prime\_K | 24.1 | 10.5 |
| P2\_N | P3prime\_E | 25.5 | 12.8 |
| P2\_Q | P2prime\_Q | 21.7 | 16.0 |
| P2\_S | P2prime\_H | 12.5 | 14.6 |
| P2\_S | P3prime\_G | 20.6 | 12.4 |
| P1\_D | P1prime\_Y | 18.1 | 30.5 |
| P1\_E | P2prime\_Q | 10.3 | 15.8 |
| P1\_Q | P1prime\_C | 16.3 | 37.1 |
| P1\_Q | P2prime\_I | 16.6 | 11.6 |
| P1\_S | P3prime\_G | 16.5 | 11.3 |
| P1prime\_Q | P2prime\_Q | 14.8 | 17.9 |
| P1prime\_V | P2prime\_T | 19.4 | 30.4 |
| P2prime\_I | P3prime\_S | 13.3 | 13.3 |
| P2prime\_K | P3prime\_E | 21.0 | 24.1 |
| P2prime\_V | P3prime\_T | 10.7 | 15.4 |

  
**PICS analysis of protease: hMMP12\_T\_1%**  
275 cleavage sites analyzed  
PICS library made with (T)rypsin, (G)luC or (C)hymotrypsin:   
Cutoff for graphic display: 2 x natural abundance  

|  |  |
| --- | --- |
| Positional occurences  (table for total and relative (in %) values) | Occurences relative to natural abundance (table) |
|  |  |
