# Supplementary material for: Active site specificity profiling datasets of matrix metalloproteinases (MMPs) 1, 2, 3, 7, 8, 9, 12, 13 and 14
Source: Data Brief. 2016 Feb 22;7:299–310. doi: 10.1016/j.dib.2016.02.036 (PMC4777984; doi:10.1016/j.dib.2016.02.036)
Supplement: Supplementary file 10 — Supplementary material [file mmc10.zip › WebPICS_hMMP13_G_1%/P1.html]

 

PICS results


|  |  |
| --- | --- |
| **P1\_A**  22 in 130 sites   16.9 %    effects > 10 perc. pnts.  (vice-versa in brackets)  P3\_A: 15.6 (12.7)   P2\_K: 21.0 (23.1)   P1prime\_Q: 17.3 (29.3)   P2prime\_T: 15.8 (38.7) |  |
  
| **P1\_C**  2 in 130 sites   1.5 %    effects > 10 perc. pnts.  (vice-versa in brackets)  P2\_H: 47.7 (31.8) |  |
  
| **P1\_G**  22 in 130 sites   16.9 %    effects > 10 perc. pnts.  (vice-versa in brackets)  P2\_V: 11.3 (27.5)   P1prime\_C: 15.0 (33.1)   P1prime\_W: 11.3 (83.1)   P2prime\_V: -10.9 (-11.9)   P3prime\_V: 22.6 (41.4) |  |
  
| **P1\_H**  5 in 130 sites   3.8 %    effects > 10 perc. pnts.  (vice-versa in brackets)  P2\_R: 30.0 (11.6)   P2\_Y: 16.9 (21.2)   P2prime\_Q: 32.3 (16.2)   P3prime\_H: 17.7 (29.5) |  |
  
| **P1\_N**  16 in 130 sites   12.3 %    effects > 10 perc. pnts.  (vice-versa in brackets)  P2\_L: 14.2 (16.3)   P2prime\_I: 23.5 (37.7)   P2prime\_Q: 11.1 (17.7)   P3prime\_N: 18.1 (32.1) |  |
  
| **P1\_P**  7 in 130 sites   5.4 %    effects > 10 perc. pnts.  (vice-versa in brackets)  P2\_L: 46.3 (23.2) |  |
  
| **P1\_Q**  8 in 130 sites   6.2 %    effects > 10 perc. pnts.  (vice-versa in brackets)  P1prime\_V: 42.3 (33.8)   P3prime\_N: 18.1 (16.0) |  |
  
| **P1\_S**  15 in 130 sites   11.5 %    effects > 10 perc. pnts.  (vice-versa in brackets)  P2\_L: 15.9 (17.1)   P2\_S: 11.5 (15.8)   P2prime\_R: 11.5 (15.8)   P3prime\_S: 17.5 (21.8) |  |
