# Supplementary material for: Active site specificity profiling datasets of matrix metalloproteinases (MMPs) 1, 2, 3, 7, 8, 9, 12, 13 and 14
Source: Data Brief. 2016 Feb 22;7:299–310. doi: 10.1016/j.dib.2016.02.036 (PMC4777984; doi:10.1016/j.dib.2016.02.036)
Supplement: Supplementary file 10 — Supplementary material [file mmc10.zip › WebPICS_hMMP13_G_1%/P1prime.html]

 

PICS results


|  |  |
| --- | --- |
| **P1prime\_C**  10 in 130 sites   7.7 %    effects > 10 perc. pnts.  (vice-versa in brackets)  P2\_V: 23.1 (25.6)   P1\_G: 33.1 (15.0)   P3prime\_K: 12.3 (12.3)   P3prime\_V: 20.8 (17.3) |  |
  
| **P1prime\_H**  5 in 130 sites   3.8 %    effects > 10 perc. pnts.  (vice-versa in brackets)  P2prime\_H: 17.7 (29.5)   P3prime\_T: 31.5 (14.4) |  |
  
| **P1prime\_I**  18 in 130 sites   13.8 %    effects > 10 perc. pnts.  (vice-versa in brackets)  P2prime\_L: 14.7 (15.6) |  |
  
| **P1prime\_L**  47 in 130 sites   36.2 %    effects > 10 perc. pnts.  (vice-versa in brackets)  P3\_P: -17.4 (-19.5) |  |
  
| **P1prime\_Q**  13 in 130 sites   10.0 %    effects > 10 perc. pnts.  (vice-versa in brackets)  P2\_R: 13.1 (13.1)   P1\_A: 29.3 (17.3) |  |
  
| **P1prime\_V**  10 in 130 sites   7.7 %    effects > 10 perc. pnts.  (vice-versa in brackets)  P1\_Q: 33.8 (42.3)   P2prime\_I: 22.3 (22.3)   P3prime\_N: 23.1 (25.6) |  |
  
| **P1prime\_W**  3 in 130 sites   2.3 %    effects > 10 perc. pnts.  (vice-versa in brackets)  P2\_A: 85.4 (13.5)   P1\_G: 83.1 (11.3)   P2prime\_K: 85.4 (13.5) |  |
