# Supplementary material for: Active site specificity profiling datasets of matrix metalloproteinases (MMPs) 1, 2, 3, 7, 8, 9, 12, 13 and 14
Source: Data Brief. 2016 Feb 22;7:299–310. doi: 10.1016/j.dib.2016.02.036 (PMC4777984; doi:10.1016/j.dib.2016.02.036)
Supplement: Supplementary file 10 — Supplementary material [file mmc10.zip › WebPICS_hMMP13_G_1%/P2.html]

 

PICS results


|  |  |
| --- | --- |
| **P2\_A**  19 in 130 sites   14.6 %    effects > 10 perc. pnts.  (vice-versa in brackets)  P3\_V: 14.0 (16.6)   P1prime\_W: 13.5 (85.4)   P2prime\_K: 22.2 (22.2) |  |
  
| **P2\_H**  3 in 130 sites   2.3 %    effects > 10 perc. pnts.  (vice-versa in brackets)  P1\_C: 31.8 (47.7)   P2prime\_T: 59.8 (19.9) |  |
  
| **P2\_K**  20 in 130 sites   15.4 %    effects > 10 perc. pnts.  (vice-versa in brackets)  P1\_A: 23.1 (21.0) |  |
  
| **P2\_L**  14 in 130 sites   10.8 %    effects > 10 perc. pnts.  (vice-versa in brackets)  P1\_N: 16.3 (14.2)   P1\_P: 23.2 (46.3)   P1\_S: 17.1 (15.9)   P2prime\_L: 15.5 (12.7) |  |
  
| **P2\_R**  13 in 130 sites   10.0 %    effects > 10 perc. pnts.  (vice-versa in brackets)  P1\_H: 11.6 (30.0)   P1prime\_Q: 13.1 (13.1)   P2prime\_R: 14.6 (17.3)   P3prime\_V: 13.9 (15.0) |  |
  
| **P2\_S**  11 in 130 sites   8.5 %    effects > 10 perc. pnts.  (vice-versa in brackets)  P1\_S: 15.8 (11.5)   P2prime\_Q: 10.5 (11.5) |  |
  
| **P2\_V**  9 in 130 sites   6.9 %    effects > 10 perc. pnts.  (vice-versa in brackets)  P1\_G: 27.5 (11.3)   P1prime\_C: 25.6 (23.1)   P2prime\_N: 16.0 (18.1)   P3prime\_K: 25.6 (23.1)   P3prime\_V: 24.1 (18.1) |  |
  
| **P2\_Y**  4 in 130 sites   3.1 %    effects > 10 perc. pnts.  (vice-versa in brackets)  P1\_H: 21.2 (16.9)   P3prime\_H: 22.7 (30.2) |  |
