# Supplementary material for: Active site specificity profiling datasets of matrix metalloproteinases (MMPs) 1, 2, 3, 7, 8, 9, 12, 13 and 14
Source: Data Brief. 2016 Feb 22;7:299–310. doi: 10.1016/j.dib.2016.02.036 (PMC4777984; doi:10.1016/j.dib.2016.02.036)
Supplement: Supplementary file 10 — Supplementary material [file mmc10.zip › WebPICS_hMMP13_G_1%/P2prime.html]

 

PICS results


|  |  |
| --- | --- |
| **P2prime\_H**  3 in 130 sites   2.3 %    effects > 10 perc. pnts.  (vice-versa in brackets)  P1prime\_H: 29.5 (17.7) |  |
  
| **P2prime\_I**  10 in 130 sites   7.7 %    effects > 10 perc. pnts.  (vice-versa in brackets)  P1\_N: 37.7 (23.5)   P1prime\_V: 22.3 (22.3)   P3prime\_H: 17.7 (59.0)   P3prime\_N: 23.1 (25.6) |  |
  
| **P2prime\_K**  19 in 130 sites   14.6 %    effects > 10 perc. pnts.  (vice-versa in brackets)  P2\_A: 22.2 (22.2)   P1prime\_W: 13.5 (85.4) |  |
  
| **P2prime\_L**  17 in 130 sites   13.1 %    effects > 10 perc. pnts.  (vice-versa in brackets)  P3\_P: -26.4 (-10.7)   P2\_L: 12.7 (15.5)   P1prime\_I: 15.6 (14.7) |  |
  
| **P2prime\_N**  8 in 130 sites   6.2 %    effects > 10 perc. pnts.  (vice-versa in brackets)  P2\_V: 18.1 (16.0)   P3prime\_V: 28.3 (18.8) |  |
  
| **P2prime\_Q**  10 in 130 sites   7.7 %    effects > 10 perc. pnts.  (vice-versa in brackets)  P2\_S: 11.5 (10.5)   P1\_H: 16.2 (32.3)   P1\_N: 17.7 (11.1) |  |
  
| **P2prime\_R**  11 in 130 sites   8.5 %    effects > 10 perc. pnts.  (vice-versa in brackets)  P2\_R: 17.3 (14.6)   P1\_S: 15.8 (11.5) |  |
  
| **P2prime\_T**  9 in 130 sites   6.9 %    effects > 10 perc. pnts.  (vice-versa in brackets)  P2\_H: 19.9 (59.8)   P1\_A: 38.7 (15.8) |  |
  
| **P2prime\_V**  20 in 130 sites   15.4 %    effects > 10 perc. pnts.  (vice-versa in brackets)  P1\_G: -11.9 (-10.9)   P3prime\_G: 10.8 (17.9)   P3prime\_S: 10.8 (17.9) |  |
