# Supplementary material for: Active site specificity profiling datasets of matrix metalloproteinases (MMPs) 1, 2, 3, 7, 8, 9, 12, 13 and 14
Source: Data Brief. 2016 Feb 22;7:299–310. doi: 10.1016/j.dib.2016.02.036 (PMC4777984; doi:10.1016/j.dib.2016.02.036)
Supplement: Supplementary file 10 — Supplementary material [file mmc10.zip › WebPICS_hMMP13_G_1%/P3.html]

 

PICS results


|  |  |
| --- | --- |
| **P3\_A**  27 in 130 sites   20.8 %    effects > 10 perc. pnts.  (vice-versa in brackets)  P1\_A: 12.7 (15.6) |  |
  
| **P3\_P**  42 in 130 sites   32.3 %    effects > 10 perc. pnts.  (vice-versa in brackets)  P1prime\_L: -19.5 (-17.4)   P2prime\_L: -10.7 (-26.4) |  |
  
| **P3\_V**  16 in 130 sites   12.3 %    effects > 10 perc. pnts.  (vice-versa in brackets)  P2\_A: 16.6 (14.0)   P3prime\_K: 23.5 (37.7) |  |
