# Supplementary material for: Active site specificity profiling datasets of matrix metalloproteinases (MMPs) 1, 2, 3, 7, 8, 9, 12, 13 and 14
Source: Data Brief. 2016 Feb 22;7:299–310. doi: 10.1016/j.dib.2016.02.036 (PMC4777984; doi:10.1016/j.dib.2016.02.036)
Supplement: Supplementary file 10 — Supplementary material [file mmc10.zip › WebPICS_hMMP13_G_1%/P3prime.html]

 

PICS results


|  |  |
| --- | --- |
| **P3prime\_G**  12 in 130 sites   9.2 %    effects > 10 perc. pnts.   (vice-versa in brackets)  P2prime\_V: 17.9 (10.8) |  |
  
| **P3prime\_H**  3 in 130 sites   2.3 %    effects > 10 perc. pnts.   (vice-versa in brackets)  P2\_Y: 30.2 (22.7)   P1\_H: 29.5 (17.7)   P2prime\_I: 59.0 (17.7) |  |
  
| **P3prime\_K**  10 in 130 sites   7.7 %    effects > 10 perc. pnts.   (vice-versa in brackets)  P3\_V: 37.7 (23.5)   P2\_V: 23.1 (25.6)   P1prime\_C: 12.3 (12.3) |  |
  
| **P3prime\_N**  9 in 130 sites   6.9 %    effects > 10 perc. pnts.   (vice-versa in brackets)  P1\_N: 32.1 (18.1)   P1\_Q: 16.0 (18.1)   P1prime\_V: 25.6 (23.1)   P2prime\_I: 25.6 (23.1) |  |
  
| **P3prime\_S**  12 in 130 sites   9.2 %    effects > 10 perc. pnts.   (vice-versa in brackets)  P1\_S: 21.8 (17.5)   P2prime\_V: 17.9 (10.8) |  |
  
| **P3prime\_T**  11 in 130 sites   8.5 %    effects > 10 perc. pnts.   (vice-versa in brackets)  P1prime\_H: 14.4 (31.5) |  |
  
| **P3prime\_V**  12 in 130 sites   9.2 %    effects > 10 perc. pnts.   (vice-versa in brackets)  P2\_R: 15.0 (13.9)   P2\_V: 18.1 (24.1)   P1\_G: 41.4 (22.6)   P1prime\_C: 17.3 (20.8)   P2prime\_N: 18.8 (28.3) |  |
