# Supplementary material for: Active site specificity profiling datasets of matrix metalloproteinases (MMPs) 1, 2, 3, 7, 8, 9, 12, 13 and 14
Source: Data Brief. 2016 Feb 22;7:299–310. doi: 10.1016/j.dib.2016.02.036 (PMC4777984; doi:10.1016/j.dib.2016.02.036)
Supplement: Supplementary file 10 — Supplementary material [file mmc10.zip › WebPICS_hMMP13_G_1%/subsite_coop.html]

 

PICS results


- # About
- # Manual
- # Analysis
- # Results

- Redraw
- Seqlogo
- Dependency
- Coop

**Potential subsite cooperativity analysis for hMMP13\_G\_1%**   
Minimum difference for subsite dependency was set to +- 10 percentage-points  
Subsite dependency was checked for all positional occurences above 1 x natural abundance  
**Subsite dependency check is restricted to P3 - P3'**

|  |  |  |  |
| --- | --- | --- | --- |
| Fixed residue | Affected residue(s) | Change (percentage-points) | Vice-Versa change |
| P3\_A | P1\_A | 12.7 | 15.6 |
| P3\_P | P1prime\_L | -19.5 | -17.4 |
| P3\_P | P2prime\_L | -10.7 | -26.4 |
| P3\_V | P2\_A | 16.6 | 14.0 |
| P3\_V | P3prime\_K | 23.5 | 37.7 |
| P2\_A | P1prime\_W | 13.5 | 85.4 |
| P2\_A | P2prime\_K | 22.2 | 22.2 |
| P2\_H | P1\_C | 31.8 | 47.7 |
| P2\_H | P2prime\_T | 59.8 | 19.9 |
| P2\_K | P1\_A | 23.1 | 21.0 |
| P2\_L | P1\_N | 16.3 | 14.2 |
| P2\_L | P1\_P | 23.2 | 46.3 |
| P2\_L | P1\_S | 17.1 | 15.9 |
| P2\_L | P2prime\_L | 15.5 | 12.7 |
| P2\_R | P1\_H | 11.6 | 30.0 |
| P2\_R | P1prime\_Q | 13.1 | 13.1 |
| P2\_R | P2prime\_R | 14.6 | 17.3 |
| P2\_R | P3prime\_V | 13.9 | 15.0 |
| P2\_S | P1\_S | 15.8 | 11.5 |
| P2\_S | P2prime\_Q | 10.5 | 11.5 |
| P2\_V | P1\_G | 27.5 | 11.3 |
| P2\_V | P1prime\_C | 25.6 | 23.1 |
| P2\_V | P2prime\_N | 16.0 | 18.1 |
| P2\_V | P3prime\_K | 25.6 | 23.1 |
| P2\_V | P3prime\_V | 24.1 | 18.1 |
| P2\_Y | P1\_H | 21.2 | 16.9 |
| P2\_Y | P3prime\_H | 22.7 | 30.2 |
| P1\_A | P1prime\_Q | 17.3 | 29.3 |
| P1\_A | P2prime\_T | 15.8 | 38.7 |
| P1\_G | P1prime\_C | 15.0 | 33.1 |
| P1\_G | P1prime\_W | 11.3 | 83.1 |
| P1\_G | P2prime\_V | -10.9 | -11.9 |
| P1\_G | P3prime\_V | 22.6 | 41.4 |
| P1\_H | P2prime\_Q | 32.3 | 16.2 |
| P1\_H | P3prime\_H | 17.7 | 29.5 |
| P1\_N | P2prime\_I | 23.5 | 37.7 |
| P1\_N | P2prime\_Q | 11.1 | 17.7 |
| P1\_N | P3prime\_N | 18.1 | 32.1 |
| P1\_Q | P1prime\_V | 42.3 | 33.8 |
| P1\_Q | P3prime\_N | 18.1 | 16.0 |
| P1\_S | P2prime\_R | 11.5 | 15.8 |
| P1\_S | P3prime\_S | 17.5 | 21.8 |
| P1prime\_C | P3prime\_K | 12.3 | 12.3 |
| P1prime\_C | P3prime\_V | 20.8 | 17.3 |
| P1prime\_H | P2prime\_H | 17.7 | 29.5 |
| P1prime\_H | P3prime\_T | 31.5 | 14.4 |
| P1prime\_I | P2prime\_L | 14.7 | 15.6 |
| P1prime\_V | P2prime\_I | 22.3 | 22.3 |
| P1prime\_V | P3prime\_N | 23.1 | 25.6 |
| P1prime\_W | P2prime\_K | 85.4 | 13.5 |
| P2prime\_I | P3prime\_H | 17.7 | 59.0 |
| P2prime\_I | P3prime\_N | 23.1 | 25.6 |
| P2prime\_N | P3prime\_V | 28.3 | 18.8 |
| P2prime\_V | P3prime\_G | 10.8 | 17.9 |
| P2prime\_V | P3prime\_S | 10.8 | 17.9 |

  
**PICS analysis of protease: hMMP13\_G\_1%**  
130 cleavage sites analyzed  
PICS library made with (T)rypsin, (G)luC or (C)hymotrypsin:   
Cutoff for graphic display: 2 x natural abundance  

|  |  |
| --- | --- |
| Positional occurences  (table for total and relative (in %) values) | Occurences relative to natural abundance (table) |
|  |  |
