# Supplementary material for: Active site specificity profiling datasets of matrix metalloproteinases (MMPs) 1, 2, 3, 7, 8, 9, 12, 13 and 14
Source: Data Brief. 2016 Feb 22;7:299–310. doi: 10.1016/j.dib.2016.02.036 (PMC4777984; doi:10.1016/j.dib.2016.02.036)
Supplement: Supplementary file 10 — Supplementary material [file mmc10.zip › WebPICS_hMMP13_T_1%/P1.html]

 

PICS results


|  |  |
| --- | --- |
| **P1\_A**  20 in 143 sites   14.0 %    effects > 10 perc. pnts.  (vice-versa in brackets)  P1prime\_I: 13.1 (15.4)   P3prime\_A: 19.7 (13.6) |  |
  
| **P1\_D**  11 in 143 sites   7.7 %    effects > 10 perc. pnts.  (vice-versa in brackets)  P1prime\_C: 12.6 (17.3)   P2prime\_H: 12.6 (17.3)   P3prime\_T: 11.9 (14.5) |  |
  
| **P1\_G**  26 in 143 sites   18.2 %    effects > 10 perc. pnts.  (vice-versa in brackets)  P2\_A: 19.6 (18.8)   P1prime\_W: 16.8 (48.5)   P2prime\_K: 17.1 (31.8)   P3prime\_A: -12.6 (-11.3)   P3prime\_G: 12.6 (12.6) |  |
  
| **P1\_H**  5 in 143 sites   3.5 %    effects > 10 perc. pnts.  (vice-versa in brackets)  P3\_I: 52.3 (23.8)   P2prime\_Q: 31.6 (13.2) |  |
  
| **P1\_N**  15 in 143 sites   10.5 %    effects > 10 perc. pnts.  (vice-versa in brackets)  P3\_A: 13.4 (10.6)   P1prime\_H: 10.5 (39.5)   P2prime\_V: 25.3 (18.1) |  |
  
| **P1\_P**  14 in 143 sites   9.8 %    effects > 10 perc. pnts.  (vice-versa in brackets)  P2\_F: 15.8 (27.7)   P2\_H: 11.5 (40.2)   P1prime\_V: 17.4 (15.2)   P2prime\_Q: 20.2 (23.5)   P2prime\_T: 15.8 (27.7)   P3prime\_H: 28.0 (35.7) |  |
  
| **P1\_Q**  6 in 143 sites   4.2 %    effects > 10 perc. pnts.  (vice-versa in brackets)  P1prime\_M: 12.5 (12.5)   P2prime\_I: 55.5 (20.8)   P3prime\_T: 43.7 (29.1) |  |
  
| **P1\_S**  13 in 143 sites   9.1 %    effects > 10 perc. pnts.  (vice-versa in brackets)  P2\_S: 32.9 (22.5)   P2prime\_L: 13.3 (12.3) |  |
  
| **P1\_W**  2 in 143 sites   1.4 %    effects > 10 perc. pnts.  (vice-versa in brackets)  P2\_H: 47.2 (23.6) |  |
