# Supplementary material for: Active site specificity profiling datasets of matrix metalloproteinases (MMPs) 1, 2, 3, 7, 8, 9, 12, 13 and 14
Source: Data Brief. 2016 Feb 22;7:299–310. doi: 10.1016/j.dib.2016.02.036 (PMC4777984; doi:10.1016/j.dib.2016.02.036)
Supplement: Supplementary file 10 — Supplementary material [file mmc10.zip › WebPICS_hMMP13_T_1%/P1prime.html]

 

PICS results


|  |  |
| --- | --- |
| **P1prime\_C**  8 in 143 sites   5.6 %    effects > 10 perc. pnts.  (vice-versa in brackets)  P3\_I: 17.3 (12.6)   P1\_D: 17.3 (12.6)   P2prime\_T: 19.4 (19.4)   P3prime\_T: 18.7 (16.6) |  |
  
| **P1prime\_H**  4 in 143 sites   2.8 %    effects > 10 perc. pnts.  (vice-versa in brackets)  P1\_N: 39.5 (10.5) |  |
  
| **P1prime\_I**  17 in 143 sites   11.9 %    effects > 10 perc. pnts.  (vice-versa in brackets)  P1\_A: 15.4 (13.1)   P3prime\_D: 13.7 (16.7) |  |
  
| **P1prime\_L**  43 in 143 sites   30.1 %    effects > 10 perc. pnts.  (vice-versa in brackets)  P3\_P: -15.0 (-11.1) |  |
  
| **P1prime\_M**  6 in 143 sites   4.2 %    effects > 10 perc. pnts.  (vice-versa in brackets)  P3\_V: 24.2 (11.2)   P2\_Q: 27.7 (20.8)   P1\_Q: 12.5 (12.5)   P2prime\_W: 30.5 (45.8)   P3prime\_S: 24.2 (11.2) |  |
  
| **P1prime\_Q**  9 in 143 sites   6.3 %    effects > 10 perc. pnts.  (vice-versa in brackets)  P2\_H: 19.4 (43.7)   P2prime\_Q: 36.0 (27.0)   P3prime\_H: 14.5 (11.9) |  |
  
| **P1prime\_V**  16 in 143 sites   11.2 %    effects > 10 perc. pnts.  (vice-versa in brackets)  P2\_F: 19.4 (38.8)   P1\_P: 15.2 (17.4)   P2prime\_I: 13.8 (13.8) |  |
  
| **P1prime\_W**  9 in 143 sites   6.3 %    effects > 10 perc. pnts.  (vice-versa in brackets)  P2\_A: 47.8 (15.9)   P1\_G: 48.5 (16.8)   P2prime\_K: 45.8 (29.4) |  |
